# Supplementary material for: Women’s experiences of the Odon Device to assist vaginal birth and participation in intrapartum research: a qualitative study in a maternity unit in the Southwest of England
Source: BMJ Open. 2021 Dec 15;11(12):e057023. doi: 10.1136/bmjopen-2021-057023 (PMC8679107; doi:10.1136/bmjopen-2021-057023)
Supplement: Supplementary data [file bmjopen-2021-057023supp001.pdf]

North Bristol NHS
NHS Trust

ASSIST
ASSISTED VAGINAL BIRTH STUDY

Patient ID_ _ _ _ Doctor ID _ _ _ _

OBSERVATIONAL DATA SCHEME

**Doctor consent verbally reconfirmed:**	**Yes** ☐	**Date:**<br>**Print:**	**Time:**<br>**Sign**
**Location**		**Operator Grade**	
**Start time of observation**		**End time of observation**	

**Who's present (circle)**	Operator<br>Trainee O&G<br>Consultant O&G	Midwife<br>Student midwife<br>MCA	Anaesthetist<br>Trainee anaesthetist<br>ODP	Scrub nurse<br>Runner<br>Other:

**Pre-delivery**	
Analgesia<br>Birthing partners<br>Consent	
**Delivery**	
Physical<br>People<br>Patient factors<br>Baby factors<br>Lighting<br>Equipment<br>Factors if BD Odon device fails?	
**Post-delivery**	
Catheter?<br>PPH?	

Observation data scheme v4 (03/06/18)

North Bristol NHS
NHS Trust

ASSIST
ASSISTED VAGINAL BIRTH STUDY

Stage	Instruction for use steps	
**Conditions of safe application**	Full Dilation<br>Head 0-+3 station<br>Cephalic presentation confirmed<br>Rupture of membranes confirmed	
	Adequate analgesia	
	Lithotomy position	
	Empty bladder	
	Re-confirm fetal position	
	Lubricate birth canal	
**Device Preparation 1**	Remove device from packaging, maintaining sterility	
	Pull back on fastening band until blue deflation line exposed	
	With fastening band still intact, generously lubricate the inside and outside of the sleeve and the cup	
	While holding the applicator handle gently slide the fastening band back to the top of the sleeve	
	Grip the applicator handle and ensure the viewing window is facing upwards	
**Device Insertion**	Fold the cup and gently insert it through the vulva	
	Check it has regained its circular shape	
	Check there is no maternal tissue trapped between the cup and the fetal head	
	With your hand on the handle of the applicator, gently push the device through the vulva	
	Use your other hand to guide the device and do not apply any force with it	
**Device Preparation 2**	Unfasten the red button	
	Open and completely remove the fastening band	
	Ensure the sleeve and applicator remain in place inside the vulva	
	Between contraction, keeping both hands away from the sleeve, continue to gently push the applicator	
	Starting at 45 degrees below the horizontal and following the curvature of the birth canal	
	Monitor progress by looking through the viewing window	
	Continue to insert the device and stop when "0" appears in the viewing window	
	Squeeze the bulb pump fully and firmly at least 8 times to inflate the cuff	
	Whilst protecting the perineum with one hand, use the other hand to completely withdraw the applicator and cuff, leaving on the sleeve in place	
	To compensate for the possible reduction in cuff pressure, squeeze the bulb pump fully and firmly two more times prior to traction	
**Delivery**	Grasp the sleeve handle, and during contractions pull gently and progressively following the J-shape of the birth canal	
	While continuing to pull gently along the J-shape of the birth canal, confirm the fetal head is descending with pulling efforts	
	If baby has not been delivered with the first pull, repeat the above three steps with any subsequent contractions	
	Once you see the blue deflation line completely, deflate the cuff by pushing on the blue deflation button	
	Pull the sleeve handle continuing to press in the deflation button following the J-shape of the birth canal	
	Continue to pull the sleeve while pressing the blue deflation button to pull the fetal head until the sleeve detaches from the head	
	Proceed to assist the birth of the baby as per normal procedure	
	Discard the disposable applicator and sleeve according to local procedure	
**Extra Steps**	Episiotomy<br>Other: need for second device	

Observation data scheme v4 (03/06/18)
