# Supplementary material for: Women’s experiences of the Odon Device to assist vaginal birth and participation in intrapartum research: a qualitative study in a maternity unit in the Southwest of England
Source: BMJ Open. 2021 Dec 15;11(12):e057023. doi: 10.1136/bmjopen-2021-057023 (PMC8679107; doi:10.1136/bmjopen-2021-057023)
Supplement: Supplementary data [file bmjopen-2021-057023supp002.pdf]

**Investigate patient experience of the study and case studies**
**Investigate patient experience of the device**
**Investigate patient view on device monitoring in trials**

**ASSIST STUDY TOPIC GUIDE - WOMEN**

**Setting:** within maternity department, Southmead hospital
**Participants:** women who have had an AVB using the Odon device

**Opening:** Interviewer will re-iterate study information, answer any questions, and re-confirm consent.

**Background:** interviewee details and icebreaker
- Enquire about the baby (name etc.)
- Enquire about their stay in hospital so far
- Enquire about any previous deliveries (NVD, assisted birth etc.)

**What has your experience been of being in the study so far?**
- Opportunity to reflect on any observations if relevant

**What was your reaction when invited to take part?**

**Probe:**
- *Views on the recruitment process?*
- *How did you react when you were asked to have your delivery observed?*
- *How did you react when you were asked to be interviewed? What was your reaction…..?*

**How was it being observed?**

**How do you feel about your birth experience?**
**How do you feel about the device that was used to help deliver your baby?**

**Probe:**
- *Would they consent to the device being used again? Or not? Why?*
- *Would they recommend it to other women in a similar situation who needed assistance to delivery their baby?*
- *Comparison to other assisted births (if applicable)*

**This is a new device, we want doctors using the device to use it at a certain standard. How do you feel about your doctor being observed delivering your baby using the device?**

**Probe:**
- *Why is it alright?*
- *Why is it not alright?*

**Closing:** interviewer checks understanding of any outstanding points, answers further questions.

Patient interview topic guide v1 (03/06/18)
