# Supplementary material for: Women’s experiences of the Odon Device to assist vaginal birth and participation in intrapartum research: a qualitative study in a maternity unit in the Southwest of England
Source: BMJ Open. 2021 Dec 15;11(12):e057023. doi: 10.1136/bmjopen-2021-057023 (PMC8679107; doi:10.1136/bmjopen-2021-057023)
Supplement: Author's manuscript [file bmjopen-2021-057023.draft_revisions.pdf]

BMJ Open is committed to open peer review. As part of this commitment we make the peer review history of every article we publish publicly available.

When an article is published we post the peer reviewers' comments and the authors' responses online. We also post the versions of the paper that were used during peer review. These are the versions that the peer review comments apply to.

The versions of the paper that follow are the versions that were submitted during the peer review process. They are not the versions of record or the final published versions. They should not be cited or distributed as the published version of this manuscript.

BMJ Open is an open access journal and the full, final, typeset and author-corrected version of record of the manuscript is available on our site with no access controls, subscription charges or pay-per-view fees (http://bmjopen.bmj.com).

If you have any questions on BMJ Open's open peer review process please email info.bmjopen@bmj.com

BMJ Open

The Odon Device to assist vaginal birth: Observations of and insights into women's experiences as narrated by health professionals and women

Journal:	*BMJ Open*
Manuscript ID	bmjopen-2021-057023
Article Type:	Original research
Date Submitted by the Author:	02-Sep-2021
Complete List of Authors:	Hotton, Emily; University of Bristol; North Bristol NHS Trust<br>Blencowe, Natalie; University of Bristol, Centre for Surgical Research, School of Social and Community Medicine<br>Lenguerrand, Erik; University of Bristol School of Clinical Science, School of Clinical Sciences, Musculoskeletal Research Unit<br>Draycott, Tim J; North Bristol NHS Trust<br>Crofts, Joanna; North Bristol NHS Trust, Women's and Children's Research<br>Wade, Julia; University of Bristol, Bristol Medical School
Keywords:	QUALITATIVE RESEARCH, OBSTETRICS, MEDICAL EDUCATION & TRAINING

SCHOLARONE™
Manuscripts

I, the Submitting Author has the right to grant and does grant on behalf of all authors of the Work (as defined in the below author licence), an exclusive licence and/or a non-exclusive licence for contributions from authors who are: i) UK Crown employees; ii) where BMJ has agreed a CC-BY licence shall apply, and/or iii) in accordance with the terms applicable for US Federal Government officers or employees acting as part of their official duties; on a worldwide, perpetual, irrevocable, royalty-free basis to BMJ Publishing Group Ltd ("BMJ") its licensees and where the relevant Journal is co-owned by BMJ to the co-owners of the Journal, to publish the Work in this journal and any other BMJ products and to exploit all rights, as set out in our licence.

The Submitting Author accepts and understands that any supply made under these terms is made by BMJ to the Submitting Author unless you are acting as an employee on behalf of your employer or a postgraduate student of an affiliated institution which is paying any applicable article publishing charge ("APC") for Open Access articles. Where the Submitting Author wishes to make the Work available on an Open Access basis (and intends to pay the relevant APC), the terms of reuse of such Open Access shall be governed by a Creative Commons licence – details of these licences and which Creative Commons licence will apply to this Work are set out in our licence referred to above.

Other than as permitted in any relevant BMJ Author's Self Archiving Policies, I confirm this Work has not been accepted for publication elsewhere, is not being considered for publication elsewhere and does not duplicate material already published. I confirm all authors consent to publication of this Work and authorise the granting of this licence.

For peer review only

BMJ Open format

The Odon Device to assist vaginal birth: Observations of and insights into women's experiences as narrated by health professionals and women

Emily J Hotton[1,2 *], Natalie S Blencowe[3,4], Erik Lenguerrand[1,2], Tim J Draycott[2], Joanna F Crofts[2], Julia Wade[5]

[1] Translational Health Sciences, University of Bristol, Bristol, UK

[2] Southmead Hospital, North Bristol NHS Trust, Bristol, UK

[3] University Hospitals Bristol NHS Foundation Trust, Bristol, UK

[4] Centre for Surgical Research, Population Health Sciences, University of Bristol, Bristol, UK

[5] Population Health Sciences, University of Bristol, Bristol, UK

*** Corresponding Author:**

Women and Children's Academic Research

The Chilterns

Maternity Unit

Southmead Hospital

Bristol

United Kingdom

BS10 5NB

Tel: 0117 414 6764

Email: emily.hotton@nhs.net

Running title: Women's experiences of the Odon Device

Abstract word count: 237

Word count: 4057

For peer review only

ABSTRACT

Objective

To investigate experiences of women who gave birth assisted by the Odon Device, an innovative device for assisted vaginal birth as reported by women, operators, and midwives and from observation data.

Design

Qualitative semi-structured interviews and observations undertaken in the context of case study work embedded in the ASSIST feasibility Study.

Setting

A tertiary referral NHS maternity unit in the Southwest of England.

Participants

Eight women, four operators and 11 midwives participated with eight observations of the assisted vaginal birth, eight interviews with women in the postnatal period, 39 interviews/reflections with operators and 19 interviews with midwives. Women in the case study research were recruited from participants in the main ASSIST Study.

Intervention

The Odon Device, an innovative device for assisted vaginal birth.

Results

For peer review only

Thirty-nine case studies were undertaken. Triangulation of multiple data sources (participant observation, interviews with women, operators, and midwives) enabled the exploration of women's experiences of the Odon Device and participation in the trial. Experiences were overwhelmingly positive. Women and midwives believed that the Odon Device provided a kinder assisted birth, whilst recognising that learning was dynamic and not all attempted uses of the device were expected to be successful.

Conclusions

Interview and observations from multiple stakeholders enabled insight into women's experiences of an innovative device for assisted vaginal birth. Applying these qualitative methods more broadly may illuminate perspectives of key stakeholders in future intrapartum intervention research and beyond.

Trial registration

ISRCTN10203171 https://doi.org/10.1186/ISRCTN10203171

Keywords

- Qualitative research
- Inventions
- Medical device
- Obstetric delivery

For peer review only

STRENGTHS AND LIMITATIONS OF THIS STUDY

- We used qualitative methodology to assess the experiences of women undergoing attempted assisted vaginal birth using the Odon Device as part of a clinical trial.
- We obtained observation and interview data from women, operators and midwives to capture a broad range of experiences.
- Our sample sizes were appropriate to provide initial feedback however, they may be limited in capturing the full nuance of opinions and attitudes.

For peer review only

Introduction

Globally 140 million babies are born each year, with an estimated 2.6 million stillbirths (50% related to intrapartum complications)[1], 2.5 million neonatal deaths (24% related to intrapartum complications)[2] and 300,000 maternal deaths (4-13% related to a complicated or prolonged second stage of labour).[3] It is suggested that a significant proportion of births complicated by a prolonged second stage of labour could be resolved by performing an assisted vaginal birth (AVB). However, despite AVB being recognised by the World Health Organization (WHO) as one of the seven critical components of basic emergency obstetric and newborn care,[4] the global rate of AVB has continued to significantly decline.

Despite this recognition of AVB as a life-saving intervention, there have been no significant developments in AVB devices since the 1950s when the introduction the ventouse/suction cup was introduced.[5] The Odon Device (Figure 1) was invented by an Argentinean Car Mechanic and then further developed by a multi-professional team of doctors, midwives and engineers as an alternative device.[6–10] The device consists of a plastic applicator and polyethylene sleeve. The sleeve is placed over the fetal head and contains a circumferential air chamber that is inflated, providing purchase for the operator to apply traction.[10]

Figure 1 The Odon Device

Courtesy and © Becton, Dickinson and Company Reprinted with permission

A core component in any evaluation of a novel device for AVB is the investigation of women's experiences. Qualitative case study methodology has previously been employed in maternity settings[11] exploring a wide range of topics from care pathways for women giving birth in Birth Centres[12] to exploring the psycho-social impact of high-risk pregnancy.[13]

For peer review only - http://bmjopen.bmj.com/site/about/guidelines.xhtml

However, there are no published examples of case study methodology being used in intrapartum studies of clinical interventions. An advantage of case study research is its ability to study phenomena across cases:[14,15] the researcher can assess similarities and differences between cases through systematic cross comparison,[15] providing reliable and rigorous results and offering an insight into the influence of context, providing deeply contextualised data.[15] The case does not have to be a person but could be an event, decision, action or object,[16] in this instance a case the event surrounding the single use of the Odon Device. This methodology is particularly useful when exploring the introduction of the Odon Device as the context includes many variables: women, allied healthcare professionals and operator. Conducting case studies in the clinical context of device use, with triangulation of data from different sources, generates greater depth of insight through the process of data triangulation, than would be available using other approaches such as serial interviews with women and operators.[17] Case studies allow for richly contextualised data as they capture multiple perspectives through interviews and observations.

This study aimed to investigate women's experiences of the Odon Device through exploring women's and health professional views and combining them with observations collected as part of a broader case study investigation embedded within a single arm feasibility study of the Odon Device for clinically indicated AVB – the ASSIST Study[10] [10,18]

Methods

For peer review only

For peer review only - http://bmjopen.bmj.com/site/about/guidelines.xhtml

119 The case study investigation included data from one or more of the following sources:
120 participant observation and interviews with women, operators, and midwives. Written
121 consent for qualitative research was provided by women concurrently at the time of
122 providing consent to participate in the ASSIST Study (either antenatally or in the latent
123 phase of labour). All Odon Device operators and midwives provided written consent to
124 participate.[18] Typical sampling, a form of purposive sampling appropriate for case study
125 methodology, was used to provide a range of operators and indications for AVB allowing for
126 the understanding of multiple perspectives from a range of variables and allowing the
127 results to be more transferable.[19,20]

128 The study was undertaken in a large tertiary referral NHS hospital in the Southwest of
129 England with approximately 6,000 births per year. It has an alongside NICU that accepts
130 babies from across the region. The Maternity Department supports women, with extensive
131 options for birthplace including: homebirth, midwife-led independent birth unit, alongside
132 midwifery unit and an obstetric led birth unit for high risk cases. Approximately 11% of all
133 births in the maternity unit were AVBs. These were all conducted on the obstetric led birth
134 unit by an obstetrician with the support of a midwife. Women were recruited (in the
135 antenatal period or during the latent phase of labour) by a research midwife and were given
136 a participant information leaflet, bespoke information video and detailed discussion with
137 the recruiting midwife.

138

139 **Data collection**

For peer review only - http://bmjopen.bmj.com/site/about/guidelines.xhtml

Participant observations commenced when the obstetrician entered the woman's room to conduct the AVB and ended when the obstetrician left the room following the birth of the baby. The researcher was a clinical obstetrician and part of the ASSIST Study Team. Contextual factors, verbal and non-verbal communication were documented on an observation schedule. The observation schedule was developed during preparatory work, prior to the commencement of the ASSIST Study (Supplementary file). All women who had an observed AVB with the Odon Device were invited to participate in an interview on the first postnatal day. The operator and midwife present at a birth using the Odon Device were invited to take part in separate interviews. Interviews were semi-structured and directed by topic guides, which were established based on pre-clinical and clinical literature.

Triangulation of data

Transcripts were created from the participant observations made during the birth. All thoughts and interpretations of the researcher were noted, often aiding the creation of specific interview questions. Any issues that required clarification from participants were highlighted during the transcription process for further discussion in subsequent interviews. Findings were successively tested and refined by the researcher as additional transcripts were analysed and explored in the interviews with participants.

Data analysis

For peer review only

Data collection and analysis ran iteratively and in parallel.[21] NVivo 12 (QSR International, Melbourne, Australia) was used to organise data from all sources as well as supporting analysis. A broadly inductive approach to analysis was used, enabling themes to be derived from the data. Triangulation of sources guided the analysis of observation data and interview transcripts from a single case to identify, outline, and organise codes. Codes were then grouped together where commonalities were noted to identify initial themes; the description and sample quotes to illustrate the theme were noted. Finally, case summaries were developed and reviewed using the constant comparative technique[22] to assess whether and how to expand or merge themes. Double coding of a proportion of interview transcripts was undertaken by JW. Commonality and variances in coding between qualitative researchers were discussed, resolved by consensus and used to further shape developing themes and sampling.

Patient and public involvement (PPI)

PPI was undertaken for all aspects of the ASSIST Study.,[10] A session on the qualitative aspects of the study was undertaken 22nd March 2018. Twelve people (pregnant women *n*=8 and their birth partners *n*=4) took part. Participants were provided with the Participant Information Leaflet, Consent Form and Interview Topic Guide to review prior to the session. Opinions on being observed during labour and interviewed post birth were specifically sought. There was unanimous agreement that the qualitative research would be accepted and welcomed by women. The opportunity to speak with an obstetrician about the birth on the first postnatal day was viewed as a positive opportunity. No substantial changes to qualitative design of the study were made as a result of this session.

For peer review only - http://bmjopen.bmj.com/site/about/guidelines.xhtml

Reflexivity

All case study research, PPI, piloting, data collection and analysis was undertaken by a medically qualified obstetrics and gynaecology trainee, Odon Device trainer and Odon Device operator for the ASSIST Study (EH). The use of the Odon Device to assist birth is highly complex and often takes only minutes to perform; without an in-depth knowledge of the device, its Instructions for Use (IFU) and AVB, subtle differences or anomalies in its use may have overlooked during data collection by a non-clinical professional. It is possible that pre-conceived ideas and suppositions were brought to the study given the researcher's specialist clinical background. To maintain rigour, reflexive comments were made after each data collection event (observation or interview) and during data analysis. These were discussed with an experienced independent qualitative researcher (JW).

Results

Presented here is the combination of observational and interview data relevant to women's experiences, derived during the ASSIST Study to evaluate the implementation of the Odon Device. Other data such as that relating to the use of the device has been analysed separately and will be published elsewhere.

The 39 case studies conducted of attempted Odon assisted births, included eight observations, eight interviews with women observed, 19 midwife interviews, 37 operator interviews and two operator reflections. Data from these sources relevant to women's

For peer review only - http://bmjopen.bmj.com/site/about/guidelines.xhtml

experiences are included in this study. Observational data collection stopped after the eighth observation as it was felt that saturation had been reached using this method and that observations had provided the knowledge and questions required to continue conducting purely operator and midwife interviews. As only observed cases had ethical approval to include interviews with women these also were completed at this stage.

Demographics and characteristics of the women included are presented in Table 1 and data collected for the 39 case studies in Table 2.

Table 1 Demographics and characteristics of women participating in the case study research compared to those in the ASSIST Study[10]

	**Case studies** n=8(%)	**ASSIST Study** n=40(%)
**Parity**		
Nulliparous	7(87.5)	34(85.0)
Multiparous	1(12.5)	6(15.0)
**Mean age in years** (mean)	28.5	29
**Ethnicity**		
White British	8(100.0)	32(80.0)
Any other white background	0(0.0)	4(10.0)
Black	0(0.0)	2(5.0)
Indian	0(0.0)	1(2.5)
Any other Asian background	0(0.0)	1(2.5)
**Mode of labour onset**		
Spontaneous	0(0.0)	6(15.0)
Induced	8(100.0)	34(85.0)
**Analgesia at time of birth**		
Epidural	4(50.0)	34(85.0)
Perineal infiltration	3(37.5)	12(30.0)
None	1(12.5)	1(2.5)
**Final mode of birth**		
Caesarean section	0(0.0)	2(5.0)
Successful birth with Odon Device	5(62.5)	19(48.0)
Assisted vaginal birth with forceps or ventouse (unsuccessful Odon Device)	3(37.5)	19(48.0)

For peer review only

Table 2 General information about the case studies (n=39)

	Data collected			
		Interviews		
Case study no.	Observation	Women	Operator	Midwife
1	X O01	X W01	X D02	X M15
2			X D03	X M18
3			X D03	X M12 X M18
4			X D03	X M10
5			X D02	X M18
6	X O02	X W02	X D02	X M19
7	X O03	X W03	X D03	X M11 X M11
8	X O04	X W04	X D02	
9			X D03	X M16
10	X O05	X W05	X D02	X M13
11	X O06	X W06	X D02	X M14
12	X O07	X W07	X D04	X M12
13			X D02	X M16
14			X D03	X M10
15	X O08	X W08	X D03	X M17
16			X D02	
17			X D04	X M20
18			X D06	
19			X D06	
20			X D04	X M11
21			X D06	
22			X D02	X M11
23			X D02	
24			X D06	
25			X D06	
26			X D02	
27			X D02	
28			X R01	
29			X D02	
30			X D06	
31			X D02	
32			X R02	
33			X D03	
34			X D03	
35			X D06	
36			X D02	
37			X D06	
38			X D06	X M11
39			X D04	

For peer review only - http://bmjopen.bmj.com/site/about/guidelines.xhtml

217

218 Observations varied in length from 33 to 68 minutes. Interviews with women lasted 7 to 10
219 minutes all were undertaken face-to-face the day after the AVB. Interviews with midwives
220 lasted 3 to 13 minutes. A total of 11 midwives were interviewed, nine clinical midwives and
221 two research midwives. Each midwife was interviewed at least once with some undertaking
222 up to four interviews. Interviews with operators lasted between 5 and 26 minutes.

223 A summary of the themes relating to the women's experiences are presented in Figure 2.

224

225 **Figure 2** Women's experiences: women's and health professional views- themes and sub-themes.

226

227 Theme one: Reasons for taking part – a different assisted birth

228 All eight women interviewed gave very similar reasons for agreeing to participate in the
229 ASSIST Study and for having the Odon Device used to assist the birth of their baby. Women's
230 experiences were predominantly positive even where Odon use had not succeeded.

231

232 A kinder and good assisted birth

233 Half the women interviewed (n=4) stated a specific aversion towards forceps assisted birth
234 as an important factor in their decision making. Midwives shared the antipathy for forceps
235 and the wish for an alternative device and an AVB that women would welcome. Aversion
236 towards forceps was linked to previous negative experiences of friends or family:

For peer review only - http://bmjopen.bmj.com/site/about/guidelines.xhtml
For peer review only

W04: *'And also the forceps, to me, looked like a torture weapon, so I was keen to give something else a go.'*

W01: *'I don't have a lot of experience of it, but I know my nephew had forceps and was very bruised and marked.'*

Women and midwives articulated similar views, the lay belief that forceps are more 'traumatic' either physically or emotionally:

M14: *'…but it definitely didn't look quite as traumatic as a forceps delivery... But I do feel like, for her, it was a nicer experience than it might have been for a forceps. I don't know if it's the word 'forceps' that traumatises ladies, but everyone seems to be terrified of them.'*

Midwives perceived that the Odon Device was kinder than other devices and described the births as 'softer', 'nicer', 'beautiful', 'easy', 'smooth' and 'gentle':

M18: *'The device use and the delivery was actually kind of one of the most beautiful deliveries of an assisted device used that I've seen.'*

This belief was also reinforced by observations from women, midwives and operators, that babies successfully delivered with the Odon Device had minimal scalp or face injury following birth. The reduction in visual marks on the baby seemed to have a big impact on the overall perception of the birth:

M20: *'These babies are coming out with no marks on them, it's fab. I think it's stunning. It's absolutely really good.'*

For peer review only

Staff strove to maintain a positive and 'normal' birth experience for women

Observations highlighted that all staff members present at time of birth, both clinical and research, strove to ensure that the birth experience was positive for women, perhaps more so than in usual clinical practice. Environmental factors seemed to stand out as creating a calm birthing experience:

> O01: *Low lighting in the room, not main lights but two spotlights providing the light to the operator only. Music on in the background was on already prior to entering the room. It was a very calm and quiet environment."*

Women's engagement led to a more positive experience

Team members perceived that birth experience was more positive for women who were felt to have really wanted a birth assisted by the Odon Device:

> M11: *'And then at that point she said, "Oh, come on doctor," she said something like, "I've got faith in you, you can do it." So, she really, really wanted him to do it. And then she said, "But if this doesn't work, I don't want forceps because of the baby's face; I'd rather have a Section."'*

The level of engagement of women and their birth partners with the Odon Device surprised the operators:

For peer review only - http://bmjopen.bmj.com/site/about/guidelines.xhtml

> D02: *'… the husband was leaning over her leg and watching exactly what I was doing and was commentating as I was applying the device… So, when I got to four, he was like, "It's on four, it's on four, it's nearly there, nearly there." I wasn't quite sure whether he was encouraging his wife or encouraging me, but it made me smile.'*

Initial altruism overtaken by genuine engagement and interest

Initial generalised altruism regarding research participation was overtaken by genuine engagement and specific interest in the study:

> W01: *'I'm quite cynical and I was like, "I'm not going to hear about this again. I just have to say yes to this." Then I read about it and actually I did find it quite interesting, so I was more than happy to sign up to it.'*

However, several women stated their surprise when they needed an AVB having assumed during the consenting process that they would not require an assisted birth.

> W06: *'It's such a low percentage, isn't it, probability-wise. So, you just, kind of, almost think, "Well, it probably won't come to this. So, might as well [consent]."'*

Theme two: 'The woman that came round, she had the right approach'

Women valued the recruitment process, this included not only the provision of information through multimedia format but also the manner of the recruiter, timing of when they were

For peer review only

For peer review only - http://bmjopen.bmj.com/site/about/guidelines.xhtml

approached and checking consent multiple times, appreciating consent as a process and not a single event.

Information video served two purposes: convenient information sharing and familiarising women with the research team

The provision of the information video allowed women to visualise and understand how the Odon Device works. They already knew what was going to happen at the time of assisted birth:

> W02: *'…we watched the video. That's what, I think, made us say yes, I suppose, the video. Just, it's quite a good little demo on the video that makes you think…'*

Women preferred this innovative multimedia approach rather than reading the information leaflets:

> W04: *'A leaflet you tend not to read it, do you? And a presentation is dull. So yes, I quite liked the video.'*

The recruitment process was a positive experience

The manner of the recruitment process was favoured by women, but it also seemed that the way it was delivered by the research team was of the upmost importance. Women appreciated the opportunity to see the team and operators in the participant video. The

For peer review only

women described how the manner of checking consent and regular interactions with the research team increased the trust in them:

> W06: *The woman that came round, she had the right approach. You don't want to be too pushy or whatever, and you need to, yes, have tact with people, and she was fine. She was good.*

Research follow-up was experienced as supportive for the women:

> W07: *'Even after delivery, just coming in to check and see that everyone's alright, honestly, I didn't find any faults.'*

Theme three: 'Everyone's situations are different'

Women's birth experiences are all very different and their perception of their birth can be influenced by many factors that are often deeply personal and context dependent. For the women interviewed, birth experiences were not affected by whether the Odon Device was successful or not or the number of healthcare professionals present at the time of birth.

> W02: *'I know, obviously, it wasn't successful, but it's not going to be successful every single time. Everyone's situations are different.' (Unsuccessful Odon birth)*

Influences of women's birth experiences are presented in Table 3. Interestingly, women focussed on outcomes for their baby and their feelings at the time of birth rather than their own birth outcomes or complications.

For peer review only - http://bmjopen.bmj.com/site/about/guidelines.xhtml

Table 3 'Everyone's situations are different' influences on birth experience.

Sub-theme	Observation and interview findings	Unsuccessful (U) or successful (S) Odon Device
Needing an assisted vaginal birth can feel overwhelming	W01: *'I did spiral into a panic…It was just my own anxiety getting on top of me at that point in time. It was more, I just have weird claustrophobia and I was like, "Oh my God, the baby is stuck. Oh my God. It's stuck in me. Oh my God. What is going to happen?" It just became really irrational.'*	U
	W07: *'But the device and that, I didn't even make… I know, obviously, it's a big thing, but I didn't even really pay any attention to it. I just thought it was a normal device, like forceps'.*	S
Unsuccessful births with the Odon device was a learning opportunity	W02: *'I know, obviously, it wasn't successful, but it's not going to be successful every single time. Everyone's situations are different. It was just out baby being difficult. It's different if every single person going didn't like it. But no, I'd, 100%, consent again.'*	U
Number of people in the room: differences between women and midwives' views	W06: *'When all the people came in and instruments and what not, I actually felt a bit of relief that I was going to get some help.'*	S
	M17: *'Yes. It does feel quite intrusive, I have to say. Having been a midwife for many years, I would probably say the majority of the births that I'm at, it is generally just myself and the couple.'*	S

For peer review only

Theme four: 'I didn't bat two eyelids about it really'

Observation was expected and acceptable to women

Women expected and presumed that there would be observations of device use in the ASSIST Study. They appreciated it was important to observe the device in real-life settings and to learn from its use:

> W02: *'You're not going to know any different. It's easy saying, "simulations, we'll do these," but until it's gone into practice on a real baby…You need to observe to see*

how it works…Or how can you, maybe, if something went wrong, fix it. How are you ever going to learn if anything…'

Observation through video recording is an extra consideration

When asked about the possibility of videoing the AVB instead of having a formal observation by a researcher, views from women were very mixed. For some it was acceptable, providing there was anonymity and that it was used for training, one stated 'no'.

Discussion

Main Findings

Qualitative research embedded within a case study investigation into the feasibility of using an innovative device for AVB was used in this study to investigate the experiences of women. Qualitative research included observations of attempted AVBs and interviews with women involved, and interviews with midwives and operators to explore women's experiences in detail. Women's experiences were overwhelmingly positive (more so than had been expected by the study team), regardless of the outcome of using the device. Noticeably study recruitment and follow-up processes valued by women and in-person observation of the birth acceptable.

Women and midwives want an alternative to forceps and ventouse. There are wide inconsistencies in the use of AVB worldwide and these are often multifactorial, relating to concerns regarding: complications of AVB and the associated litigation,[23] limited availability

For peer review only

For peer review only - http://bmjopen.bmj.com/site/about/guidelines.xhtml

of devices in certain settings, public perception,[24] and insufficient training in devices or complex interventions.[25] This research highlighted a clear maternal aversion towards forceps and a wish for a kinder AVB. There was genuine engagement in research and positive feedback from midwives regarding their perception of the device. Women appreciated the recruitment process and the methodology used for information sharing, especially via the participant information video. Providing the women with an information video proved vital in aiding their understanding of the device as well as being a welcome format for receiving information. Women understood the need for qualitative research and expressed an awareness of the need to observe and learn from each assisted birth. Unsuccessful births with the Odon Device were not viewed by women as a failure, rather a learning opportunity for the research team. There was some reservation from midwives regarding the number of people present in the room for an AVB with the Odon Device, especially with formal observation however, it was viewed as acceptable if women were happy. As it was the first time the Odon Device had been used in clinically indicated cases[10], there was an expectation from women for their births to be formally observed. When asked whether they would agree to their AVBs being video-recorded the views from women were very varied.

Strengths and Limitations

This is the first study to undertake an in-depth qualitative exploration of the Odon Device for clinically indicated AVBs. Capturing the experiences of women is vital in assessing the device in clinical trials. Conducting case studies on labour ward enabled researchers to gather a ‘genuine’ representation of experience within the environment in which the device

For peer review only - http://bmjopen.bmj.com/site/about/guidelines.xhtml

is used that might not have been achieved using alternative approaches.[17] Another key advantage of gathering data from multiple sources (participant observation and interviews) is the opportunity for triangulation of a wealth of data. Data amalgamation (within cases) and constant cross case comparison (within and across cases) improved the validity of the study findings. A further strength is that where there are indications that saturation has been reached, there is an opportunity for one form of data collection to be dropped, applied here to observation data. Reporting was undertaken following the Standards for Reporting Qualitative Research[26] (S2).

Despite these advantages, there are limitations to qualitative case study research in this instance. There were only eight case studies that involved observation and interviews with women, because data saturation was reached from observations. This limitation raises the possibility that findings are less generalisable. Furthermore, the interviews with women were short. There are several reasons as to why this may be the case. Firstly, women were only asked a very small number of questions specifically relating to their birth experiences. Operators and midwives however were asked questions not only on experience but device use, technique, design and trial methodology. Additionally, women were interviewed at day one postnatal, when likely fatigued. This was discussed during the PPI sessions and women agreed that interviewing women in hospital close to the birth was more suitable that at home, later as women were aware that their memory of their birth experience may not be reliable. The case studies were undertaken by a specialist trainee in obstetrics and gynaecology meaning that pre-conceptions and existing knowledge may have influenced the collection and interpretation of the data. However, at the time of commencing the case studies the researcher was naïve to the use of the Odon Device in the clinical setting. Lastly,

For peer review only

operators may have changed their behaviours during observations, perhaps not reflecting their real-life practice. It is possible that there was response bias as women knew that the researcher was a clinician in the trial. However, this was discussed during PPI and felt to instead be a key aspect of the study for women. Women and their partners were keen to discuss their birth with a clinician who knew the device, feeling it was an essential aspect of the research process.

Interpretation

With novel devices being tested in clinical trials, it is vital that the experiences of key stakeholders are explored. Having an innovative device for AVB that has high clinical efficacy is not useful if women find it unacceptable. Our findings suggest that women are willing to accept an alternative device for AVB, especially one that is perceived as kinder to their babies. Women commended the use of video during the recruitment processes and expressed a general enthusiasm for intrapartum research.

This study demonstrates that qualitative case study methodology enables the detailed exploration of experiences and views of women and healthcare professionals in intrapartum research and that the methods (especially observation) were acceptable and expected by women.

Conclusion

For peer review only - http://bmjopen.bmj.com/site/about/guidelines.xhtml

When introducing or implementing a novel device into clinical use it is vital that the experiences and opinions of stakeholders are considered. Case studies provide a way of exploring these views in detail by integrating data from multiple sources, providing a comprehensive and authentic exploration. Case study methodology is used widely in other avenues of research however it has not yet been adopted in implementation research during device or surgical trials. Successful completion of case studies on labour ward for an intrapartum intervention has provided methodology that can be transferred to intervention delivery and design of novel devices. Furthermore, this methodology could be used within the broader context of randomised controlled trials for novel devices both within obstetrics and beyond. They have the potential to inform not only the conduct of trials but also provide insight into how stakeholders perceive an intervention. These perceptions can help shape and design future recruitment strategies, information provision and intervention delivery. The use of case study methodology must be further tested in alternative settings and specialties.

Acknowledgements

The authors would like to thank all the women who agreed to take part in this research and all the maternity staff who enabled to safe completion of the ASSIST Study.

Competing interests

For peer review only

EL is a member of University of Bristol (UoB) and part of his salary is paid by PROMPT Maternity Foundation (PMF); PMF has received funding from a Saving Lives at Birth award via a subcontract from Becton, Dickinson and Company (BD) to conduct preclinical simulation studies of the Odon Device, these funds have been used towards the salary of TJD and JFC when undertaking the simulations studies. All other authors report no competing interests.

Author contributions

EJH, NSB and JW developed the concept for the qualitative research. EJH performed all data collection and analysis with co-coding performed by JW. EJH wrote the initial draft of the manuscript. All authors reviewed and approved the final manuscript.

Ethical approval

The research was approved by South Central–Berkshire REC, UK on 3rd September 2018 (18/SC/0344), the MHRA on 9th August 2018 and the HRA on 3rd September 2018.

Data sharing statement: Deidentified data may be available on request.

Funding

For peer review only

This research was supported by the Bill & Melinda Gates Foundation [OPP1184825/INV-010180]. The Foundation had no role in study design, planning, conduct, analysis or publication production.

For peer review only

References

1. Lawn JE, Blencowe H, Waiswa P, Amouzou A, Mathers C, Hogan D, et al. Stillbirths: rates, risk factors, and acceleration towards 2030. Lancet. 2016;387(10018):587–603.

2. (UNIGME) UNIG for CME. Levels & Trends in Child Mortality: Report 2019, Estimates developed by the United Nations Inter-agency Group for Child Mortality Estimation. 2019.

3. Organisation WH. Trends in maternal mortality: 2000 to 2017: estimates by WHO, UNICEF, UNFPA, World Bank Group and the United Nations Population Division. [Internet]. 2019. Available from: https://www.unfpa.org/featured-publication/trends-maternal-mortality-2000-2017

4. Organization WH, UNICEF, AMDD, UNFPA. Monitoring emergency obstet- ric care: a handbook [Internet]. WHO; 2009 [cited 2020 Dec 14]. Available from: http://whqlibdoc.who.int/publications/ 2009/9789241547734_eng.pdf?ua=1

5. Malmstrom T. The vacuum extractor, an obstetrical instrument. I. Acta obstetricia et gynecologica Scandinavica Supplement. 1957;36(suppl 3):5–50.

6. O'Brien SM, Mouser A, Odon JE, Winter C, Draycott TJ, Sumitro T, et al. Design and development of the BD Odon DeviceTM : a human factors evaluation process. BJOG: An International Journal of Obstetrics & Gynaecology. 2017;124 Suppl 4:35–43.

7. O'Brien SM, Winter C, Burden CA, Boulvain M, Draycott TJ, Crofts JF. Fetal head position and perineal distension associated with the use of the BD Odon Device™ in operative vaginal birth: a simulation study. BJOG: An International Journal of Obstetrics & Gynaecology. 2017 Sep;124(11):10–8.

8. O'Brien SM, Winter C, Burden CA, Boulvain M, Draycott TJ, Crofts JF. Pressure and traction on a model fetal head and neck associated with the use of forceps, Kiwi™ ventouse and the BD Odon Device™ in operative vaginal birth: a simulation study. BJOG: An International Journal of Obstetrics & Gynaecology. 2017 Sep 1;124(11):19–25.

9. Lange C de, Saugstad OD, Solberg R. Assessment of cerebral perfusion with contrast-enhanced ultrasound during constriction of the neck mimicking malposition of the BD Odon Device™: a study in newborn piglets. BJOG: An International Journal of Obstetrics & Gynaecology. 2017 Sep;124 Suppl 4(4 Pt 1):26–34.

10. Hotton EJ, Lenguerrand E, Alvarez M, O'Brien S, Draycott TJ, Crofts JF, et al. Outcomes of the novel Odon Device in indicated operative vaginal birth. Am J Obstet Gynecol. 2020;

11. Atchan M, Davis D, Foureur M. A methodological review of qualitative case study methodology in midwifery research. Journal of advanced nursing. 2016;72(10):2259–71.

For peer review only

For peer review only - http://bmjopen.bmj.com/site/about/guidelines.xhtml

12. Bick DE, Rycroft-Malone J, Fontenla M. A case study evaluation of implementation of a care pathway to support normal birth in one English birth centre: anticipated benefits and unintended consequences. BMC pregnancy and childbirth. 2009;9:47.

13. Sittner BJ, DeFrain J, Hudson DB. Effects of high-risk pregnancies on families. MCN, The American Journal of Maternal/Child Nursing. 2005;30(2):121–6.

14. Yin RK. Case Study Research. 5th ed. SAGE Publications Ltd; 2014. (SAGE Publications Ltd).

15. Baxter P, Jack S. Qualitative Case Study Methodology: Study Design and Implementation for Novice Researchers. The Qualitative Report. 2008;13(4):544–59.

16. Bruni A. Shadowing Software and Clinical Records: On the Ethnography of Non-Humans and Heterogeneous Contexts. Organization [Internet]. 2005;12(3):357–78. Available from: http://journals.sagepub.com/doi/10.1177/1350508405051272

17. Rowley J. Using case studies in research. Management Res News. 2002;Volume 25(Issue 1):16–27.

18. O'Brien S, Hotton EJ, Lenguerrand E, Winter C, Draycott TJ, Crofts JF, et al. The ASSIST Study - The BD Odon Device for assisted vaginal birth: a safety and feasibility study. Trials. 2019;20(1).

19. Koerber A, McMichael L. Qualitative Sampling Methods. Journal of Business and Technical Communication. 2008 Oct;22(4):454–73.

20. Reeves S, Kuper A, Hodges BD. Qualitative research methodologies: ethnography. BMJ (Clinical research ed). 2008;337:a1020.

21. Braun V, Clarke V. Using thematic analysis in psychology. Qualitative Research in Psychology. 2006;3(2):77–101.

22. Brink E, Dellve L, Hallberg U, Abrahamsson KH, Klingberg G, Wentz K. Constructing grounded theory. A practical guide through qualitative analysis. Int J Qual Stud Heal. 2006;1(3).

23. Murphy DJ. Medico-legal considerations and operative vaginal delivery. Best Pract Res Clin Obstetrics Gynaecol. 2019;56:114–24.

24. J F. Why do doctors still use forceps when they killed out baby? Daily Mail [Internet]. 2010 Feb 22;1–36. Available from: https://www.dailymail.co.uk/health/article-1253013/Forceps-killed-baby-doctors-using-them.html

25. Gale A, Siassakos D, Attilakos G, Winter C, Draycott T. Operative vaginal birth: better training for better outcomes. BJOG: An International Journal of Obstetrics & Gynaecology. 2014;121(5):643–4.

For peer review only

For peer review only - http://bmjopen.bmj.com/site/about/guidelines.xhtml

541 26. O'Brien BC, Harris IB, Beckman TJ, Reed DA, Cook DA. Standards for Reporting
542 Qualitative Research: A Synthesis of Recommendations. Acad Med. 2014;89(9):1245–51.

543

For peer review only

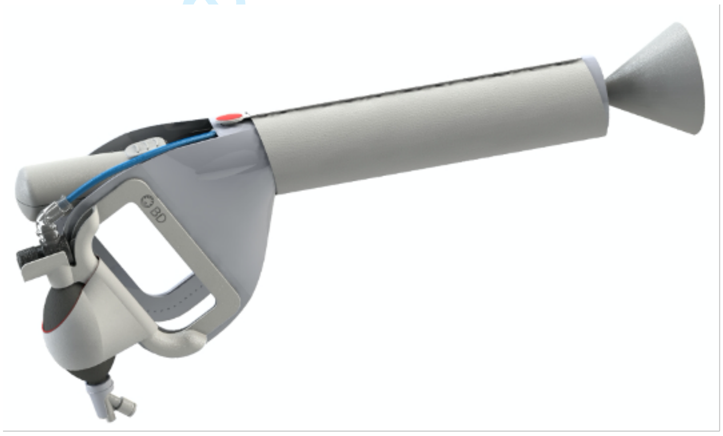

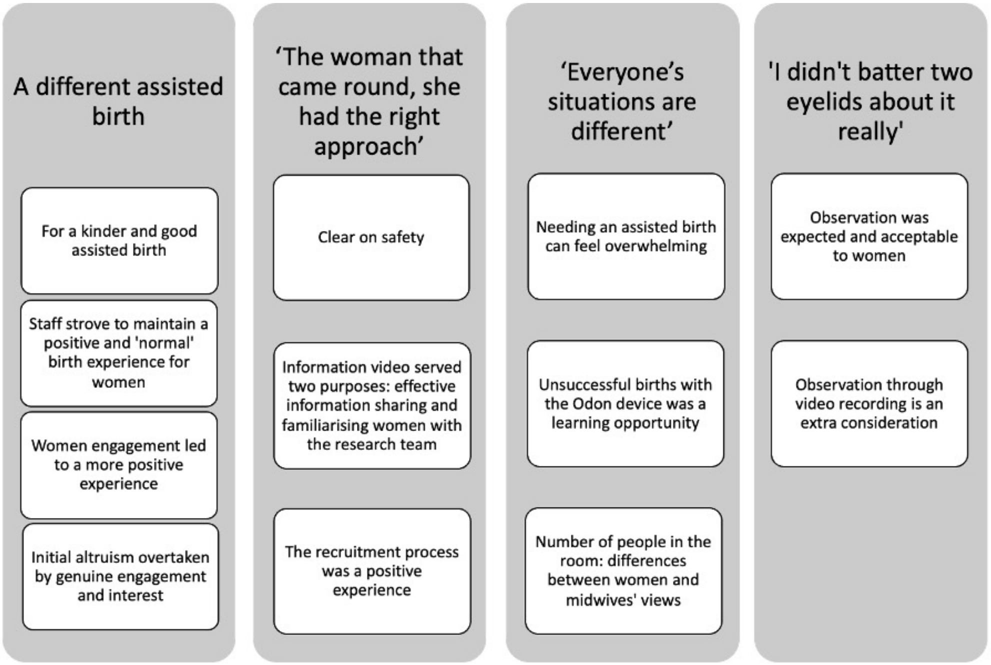

Women's experiences: women's and health professional views- themes and sub-themes

159x105mm (144 x 144 DPI)

North Bristol NHS
NHS Trust

ASSIST
ASSISTED VAGINAL BIRTH STUDY

: ’ ()$+(,;<=,=,=,=,,,,,,<* . (*%,;<,=,=,=,=

OBSERVATIONAL DATA SCHEME

**Doctor consent verbally reconfirmed:**	**Yes ☐**	**Date:**<br>**Print:**		**Time:**<br>**Sign**	
**Location**		**Operator Grade**			
**Start time of observation**		**End time of observation**			
**Who’s present (circle)**	Operator<br>Trainee O&G<br>Consultant O&G	Midwife<br>Student midwife<br>MCA	Anaesthetist<br>Trainee anaesthetist<br>ODP	Scrub nurse<br>Runner<br>Other:	

: %$>-$?)&$%@,	,
Analgesia<br>Birthing partners<br>Consent	, , , , , , , , ,
<$?)&$%@,	,
Physical<br>People<br>Patient factors<br>Baby factors<br>Lighting<br>Equipment<br>Factors if BD Odon device fails?<br>, , , ,	, , , , , , , , , , , , , , , ,
: *#(>-$?)&$%@,	,
Catheter?<br>PPH?<br>, , , , , ,	,

For peer review only - http://bmjopen.bmj.com/site/about/guidelines.xhtml
For peer review only

North Bristol NHS
NHS Trust

ASSIST
ASSISTED VAGINAL BIRTH STUDY

Stage	Instruction for use steps	
**Conditions of safe application**	Full Dilation<br>Head 0-+3 station<br>Cephalic presentation confirmed<br>Rupture of membranes confirmed	
	Adequate analgesia	
	Lithotomy position	
	Empty bladder	
	Re-confirm fetal position	
	Lubricate birth canal	
**Device Preparation 1**	Remove device from packaging, maintaining sterility	
	Pull back on fastening band until blue deflation line exposed	
	With fastening band still intact, generously lubricate the inside and outside of the sleeve and the cup	
	While holding the applicator handle gently slide the fastening band back to the top of the sleeve	
	Grip the applicator handle and ensure the viewing window is facing upwards	
**Device Insertion**	Fold the cup and gently insert it through the vulva	
	Check it has regained its circular shape	
	Check there is no maternal tissue trapped between the cup and the fetal head	
	With your hand on the handle of the applicator, gently push the device through the vulva	
	Use your other hand to guide the device and do not apply any force with it	
**Device Preparation 2**	Unfasten the red button	
	Open and completely remove the fastening band	
	Ensure the sleeve and applicator remain in place inside the vulva	
	Between contraction, keeping both hands away from the sleeve, continue to gently push the applicator	
	Starting at 45 degrees below the horizontal and following the curvature of the birth canal	
	Monitor progress by looking through the viewing window	
	Continue to insert the device and stop when “0” appears in the viewing window	
	Squeeze the bulb pump fully and firmly at least 8 times to inflate the cuff	
	Whilst protecting the perineum with one hand, use the other hand to completely withdraw the applicator and cuff, leaving on the sleeve in place	
	To compensate for the possible reduction in cuff pressure, squeeze the bulb pump fully and firmly two more times prior to traction	
**Delivery**	Grasp the sleeve handle, and during contractions pull gently and progressively following the J-shape of the birth canal	
	While continuing to pull gently along the J-shape of the birth canal, confirm the fetal head is descending with pulling efforts	
	If baby has not been delivered with the first pull, repeat the above three steps with any subsequent contractions	
	Once you see the blue deflation line completely, deflate the cuff by pushing on the blue deflation button	
	Pull the sleeve handle continuing to press in the deflation button following the J-shape of the birth canal	
	Continue to pull the sleeve while pressing the blue deflation button to pull the fetal head until the sleeve detaches from the head	
	Proceed to assist the birth of the baby as per normal procedure	
	Discard the disposable applicator and sleeve according to local procedure	
**Extra Steps**	Episiotomy<br>Other: need for second device	

,

,

For peer review only

For peer review only - http://bmjopen.bmj.com/site/about/guidelines.xhtml

Table S3 Standards for Reporting Qualitative Research (SRQR)

No.	Topic	Page number in manuscript
	**Title and abstract**	
S1	Title	1
S2	Abstract	2-3
	**Introduction**	
S3	Problem formulation	5-6
S4	Purpose or research question	6
	**Methods**	
S5	Qualitative approach and research paradigm	7
S6	Research characteristics and reflexivity	10
S7	Context	7
S8	Sampling strategy	7
S9	Ethical issues pertaining to human subjects	11
S10	Data collection methods	8
S11	Data collection instruments and technologies	8
S12	Units of study	10-11
S13	Data processing	7-9
S14	Data analysis	9
S15	Techniques to enhance trustworthiness	8-10
	**Results/findings**	
S16	Synthesis and interpretation	10-18
S17	Links to empirical data	10-18
	**Discussion**	
S18	Integration with prior work, implications, transferability, and contribution(s) to the field	18-22
S19	Limitations	20
	**Other**	
S20	Conflicts of interest	23
S21	Funding	24

For peer review only

No.	Topic	Item
	**Title and abstract**	
S1	Title	Concise description of the nature and topic of the study Identifying the study as qualitative or indicating the approach (e.g., ethnography, grounded theory) or data collection methods (e.g., interview, focus group) is recommended
S2	Abstract	Summary of key elements of the study using the abstract format of the intended publication; typically includes background, purpose, methods, results, and conclusions
	**Introduction**	
S3	Problem formulation	Description and significance of the problem/phenomenon studied; review of relevant theory and empirical work; problem statement
S4	Purpose or research question	Purpose of the study and specific objectives or questions
	**Methods**	
S5	Qualitative approach and research paradigm	Qualitative approach (e.g., ethnography, grounded theory, case study, phenomenology, narrative research) and guiding theory if appropriate; identifying the research paradigm (e.g., postpositivist, constructivist/ interpretivist) is also recommended; rationale[b]
S6	Researcher characteristics and reflexivity	Researchers' characteristics that may influence the research, including personal attributes, qualifications/experience, relationship with participants, assumptions, and/or presuppositions; potential or actual interaction between researchers' characteristics and the research questions, approach, methods, results, and/or transferability
S7	Context	Setting/site and salient contextual factors; rationale[b]
S8	Sampling strategy	How and why research participants, documents, or events were selected; criteria for deciding when no further sampling was necessary (e.g., sampling saturation); rationale[b]
S9	Ethical issues pertaining to human subjects	Documentation of approval by an appropriate ethics review board and participant consent, or explanation for lack thereof; other confidentiality and data security issues
S10	Data collection methods	Types of data collected; details of data collection procedures including (as appropriate) start and stop dates of data collection and analysis, iterative process, triangulation of sources/methods, and modification of procedures in response to evolving study findings; rationale[b]
S11	Data collection instruments and technologies	Description of instruments (e.g., interview guides, questionnaires) and devices (e.g., audio recorders) used for data collection; if/how the instrument(s) changed over the course of the study
S12	Units of study	Number and relevant characteristics of participants, documents, or events included in the study; level of participation (could be reported in results)
S13	Data processing	Methods for processing data prior to and during analysis, including transcription, data entry, data management and security, verification of data integrity, data coding, and anonymization/deidentification of excerpts
S14	Data analysis	Process by which inferences, themes, etc., were identified and developed, including the researchers involved in data analysis; usually references a specific paradigm or approach; rationale [b]
S15	Techniques to enhance trustworthiness	Techniques to enhance trustworthiness and credibility of data analysis (e.g., member checking, audit trail, triangulation); rationale[b]
	**Results/findings**	
S16	Synthesis and interpretation	Main findings (e.g., interpretations, inferences, and themes); might include development of a theory or model, or integration with prior research or theory
S17	Links to empirical data	Evidence (e.g., quotes, field notes, text excerpts, photographs) to substantiate analytic findings
	**Discussion**	
S18	Integration with prior work, implications, transferability, and contribution(s) to the field	Short summary of main findings; explanation of how findings and conclusions connect to, support, elaborate on, or challenge conclusions of earlier scholarship; discussion of scope of application/ generalizability; identification of unique contribution(s) to scholarship in a discipline or field
S19	Limitations	Trustworthiness and limitations of findings
	**Other**	
S20	Conflicts of interest	Potential sources of influence or perceived influence on study conduct and conclusions; how these were managed
S21	Funding	Sources of funding and other support; role of funders in data collection, interpretation, and reporting

[a]The authors created the SRQR by searching the literature to identify guidelines, reporting standards, and critical appraisal criteria for qualitative research; reviewing the reference lists of retrieved sources; and contacting experts to gain feedback. The SRQR aims to improve the transparency of all aspects of qualitative research by providing clear standards for reporting qualitative research.

[b]The rationale should briefly discuss the justification for choosing that theory, approach, method, or technique rather than other options available, the assumptions and limitations implicit in those choices, and how those choices influence study conclusions and transferability. As appropriate, the rationale for several items might be discussed together.

ACADEMIC MEDICINE

BMJ Open

Women's experiences of the Odon Device to assist vaginal birth and participation in intrapartum research: a qualitative study in a maternity unit in the Southwest of England

Journal:	*BMJ Open*
Manuscript ID	bmjopen-2021-057023.R1
Article Type:	Original research
Date Submitted by the Author:	28-Oct-2021
Complete List of Authors:	Hotton, Emily; University of Bristol; North Bristol NHS Trust<br>Blencowe, Natalie; University of Bristol, Centre for Surgical Research, School of Social and Community Medicine<br>Lenguerrand, Erik; University of Bristol School of Clinical Science, School of Clinical Sciences, Musculoskeletal Research Unit<br>Draycott, Tim J; North Bristol NHS Trust<br>Crofts, Joanna; North Bristol NHS Trust, Women's and Children's Research<br>Wade, Julia; University of Bristol, Bristol Medical School
**Primary Subject Heading**:	Obstetrics and gynaecology
Secondary Subject Heading:	Evidence based practice, Health services research, Qualitative research, Research methods
Keywords:	QUALITATIVE RESEARCH, OBSTETRICS, MEDICAL EDUCATION & TRAINING

SCHOLARONE™
Manuscripts

I, the Submitting Author has the right to grant and does grant on behalf of all authors of the Work (as defined in the below author licence), an exclusive licence and/or a non-exclusive licence for contributions from authors who are: i) UK Crown employees; ii) where BMJ has agreed a CC-BY licence shall apply, and/or iii) in accordance with the terms applicable for US Federal Government officers or employees acting as part of their official duties; on a worldwide, perpetual, irrevocable, royalty-free basis to BMJ Publishing Group Ltd ("BMJ") its licensees and where the relevant Journal is co-owned by BMJ to the co-owners of the Journal, to publish the Work in this journal and any other BMJ products and to exploit all rights, as set out in our licence.

The Submitting Author accepts and understands that any supply made under these terms is made by BMJ to the Submitting Author unless you are acting as an employee on behalf of your employer or a postgraduate student of an affiliated institution which is paying any applicable article publishing charge ("APC") for Open Access articles. Where the Submitting Author wishes to make the Work available on an Open Access basis (and intends to pay the relevant APC), the terms of reuse of such Open Access shall be governed by a Creative Commons licence – details of these licences and which Creative Commons licence will apply to this Work are set out in our licence referred to above.

Other than as permitted in any relevant BMJ Author's Self Archiving Policies, I confirm this Work has not been accepted for publication elsewhere, is not being considered for publication elsewhere and does not duplicate material already published. I confirm all authors consent to publication of this Work and authorise the granting of this licence.

For peer review only

Women's experiences of the Odon Device to assist vaginal birth and participation in intrapartum research: a qualitative study in a maternity unit in the Southwest of England

Emily J Hotton[1,2 *], Natalie S Blencowe[3,4], Erik Lenguerrand[1,2], Tim J Draycott[2], Joanna F Crofts[2], Julia Wade[5]

[1] Translational Health Sciences, University of Bristol, Bristol, UK

[2] Southmead Hospital, North Bristol NHS Trust, Bristol, UK

[3] University Hospitals Bristol NHS Foundation Trust, Bristol, UK

[4] Centre for Surgical Research, Population Health Sciences, University of Bristol, Bristol, UK

[5] Population Health Sciences, University of Bristol, Bristol, UK

*** Corresponding Author:**

Women and Children's Academic Research

The Chilterns

Maternity Unit

Southmead Hospital

Bristol

United Kingdom

BS10 5NB

Tel: 0117 414 6764

Email: emily.hotton@nhs.net

Running title: Women's experiences of the Odon Device

Abstract word count: 243

Word count: 4520

ABSTRACT

Objective

For peer review only

To investigate women's experiences of having a birth assisted by the Odon Device (an innovative device for assisted vaginal birth) and participation in intrapartum research.

Design

Qualitative semi-structured interviews and observations undertaken in the context of case study work embedded in the ASSIST feasibility Study.

Setting

A tertiary referral NHS maternity unit in the Southwest of England, between 8th October 2018 and 26th January 2019.

Participants

Eight women, four operators and 11 midwives participated with eight observations of the assisted vaginal birth, eight interviews with women in the postnatal period, 39 interviews/reflections with operators and 19 interviews with midwives. Women in the case study research were recruited from participants in the main ASSIST Study.

Intervention

The Odon Device, an innovative device for assisted vaginal birth.

Results

Thirty-nine case studies were undertaken. Triangulation of data sources (participant observation, interviews with women, operators, and midwives) enabled the exploration of women's experiences of the Odon Device and recruitment in the intrapartum trial. Experiences were overwhelmingly positive. Women were motivated to take part by a wish

For peer review only

for a kinder birth, and because they perceived both the recruitment and research processes (including observation) to be highly acceptable, regardless of whether the Odon assisted birth was successful or not.

Conclusions

Interviews and observations from multiple stakeholders enabled insight into women's experiences of an innovative device for assisted vaginal birth. Applying these qualitative methods more broadly may illuminate perspectives of key stakeholders in future intrapartum intervention research and beyond.

Trial registration

ASSIST Study registration: ISRCTN10203171 **https://doi.org/10.1186/ISRCTN10203171**

Keywords

- Qualitative research
- Inventions
- Medical device
- Obstetric delivery

For peer review only

For peer review only - http://bmjopen.bmj.com/site/about/guidelines.xhtml

STRENGTHS AND LIMITATIONS OF THIS STUDY

- We used qualitative methodology to assess the experiences of women undergoing attempted assisted vaginal birth using the Odon Device as part of a clinical trial.
- We obtained observation and interview data from women, operators and midwives to capture a broad range of experiences.
- Our sample sizes were appropriate to provide initial insights however, they may be limited in capturing the full nuance of opinions and attitudes.

For peer review only

Introduction

Globally 140 million babies are born each year, with an estimated 2.6 million stillbirths (50% related to intrapartum complications)[1], 2.5 million neonatal deaths (24% related to intrapartum complications)[2] and 300,000 maternal deaths (4-13% related to a complicated or prolonged second stage of labour).[3] It is suggested that a significant proportion of births complicated by a prolonged second stage of labour could be resolved by performing an assisted vaginal birth (AVB). However, despite AVB being recognised by the World Health Organization (WHO) as one of the seven critical components of basic emergency obstetric and newborn care,[4] the global rate of AVB has continued to decline significantly. Declining rates of AVB have been attributed to concerns regarding: complications of AVB and the associated litigation,[5] limited availability of devices in certain settings, public perception,[6] and insufficient training in device use or complex interventions.[7]

AVB is associated with better maternal and neonatal outcomes than Caesarean section at full dilatation, when performed by a skilled operator in an appropriate setting.[8] Specifically, women having an AVB have reduced rate of postpartum haemorrhage and reduced length of stay, and babies are less likely to be admitted to the neonatal intensive care unit (NICU).[8]

AVB is almost always performed in an emergency setting, limiting time for information provision.[9] Nevertheless, it is important to ensure that women and their birth partners, feel informed, empowered and supported during their birth experience.[10,11] A recent review suggests that AVBs can be seen as positive by women if they receive good communication from the team and feel involved in decision-making.[12] This is despite the general negative public perception of AVB, particularly forceps.

For peer review only

The consequences of a poorly performed AVB can be devastating and long lasting for mother, baby and operator. Comprehensive training, appropriate supervision and support can drastically improve outcomes and patient and operator experience.[13–16]

Despite recognition of AVB as a life-saving intervention, there have been no significant developments in AVB devices since the 1950s when the ventouse/suction cup was introduced.[17] The Odon Device (Figure 1) was invented by an Argentinean Car Mechanic and then further developed by a multi-professional team of doctors, midwives and engineers as an alternative device.[18–22] The device consists of a plastic applicator and polyethylene sleeve. The sleeve is placed over the fetal head and contains a circumferential air chamber that is inflated, providing purchase for the operator to apply traction.[22]

Figure 1 The Odon Device

Courtesy and © Becton, Dickinson and Company Reprinted with permission

A core component in any evaluation of a novel device for AVB is the investigation of women's experiences. Qualitative case study methodology has previously been employed in maternity settings[23] exploring a range of topics from care pathways for women giving birth in Birth Centres[24] to the psycho-social impact of high-risk pregnancy.[25] However, there are no published examples of case study methodology being used in intrapartum studies of clinical interventions. There is limited research exploring the experiences of women regarding recruitment processes during intrapartum research;[26] those that exist report that gaining consent for intrapartum research can be challenging, especially if women are in pain or fatigued.[27]

For peer review only - http://bmjopen.bmj.com/site/about/guidelines.xhtml

This study investigated women's experiences of the Odon Device and participation in intrapartum research through exploring women's and health professional views and combining them with observations collected as part of a broader case study investigation embedded within a single arm feasibility study of the Odon Device for clinically indicated AVB – the ASSIST Study.[22,2822,28]

Methods

Research design

Qualitative case study methodology enables systematic study of phenomena across cases, enabling identification of similarities and differences and resulting in detailed, contextualised insights. The case does not have to be a person but could be an event, decision, action or object.[29] In this study a case was the event surrounding the single use of the Odon Device. This methodology is particularly useful when exploring the introduction of the Odon Device as the context includes multiple variables: a range of reasons for requiring AVB, women, allied healthcare professionals and operator.

Study context

The study was undertaken in a large tertiary referral NHS hospital in the Southwest of England with approximately 6,000 births per year. It has an alongside NICU that accepts babies from across the region. The Maternity Department supports women, with extensive options for birthplace including: homebirth, midwife-led independent birth unit, alongside midwifery unit and an obstetric-led birth unit for high risk cases. Approximately 11% of all

For peer review only

births in the maternity unit were AVBs. These were all conducted on the obstetric-led birth unit by an obstetrician with the support of a midwife.

Participants

Women were eligible to take part in ASSIST if they were: aged 18 years or older, may require an AVB with a live, singleton pregnancy with negative antenatal screening to blood-bourne viruses.[22] Women were recruited to the ASSIST study (in the antenatal period or during the latent phase of labour) by a research midwife and were given a participant information leaflet, bespoke information video and detailed discussion with the recruiting midwife.[28] Written consent for the integrated qualitative research reported here was provided by women concurrently at the time of providing consent to participate in the ASSIST Study (either antenatally or in the latent phase of labour). All Odon Device operators and midwives provided written consent to participate.[28]

Sampling

Typical sampling, a form of purposive sampling appropriate for case study methodology, was used to include a range of indications for AVB and a range of operators allowing for the understanding of multiple perspectives across a range of variables and for results to be more transferable.[30,31]

Patient and public involvement (PPI)

PPI was undertaken for all aspects of the ASSIST Study.[22] A session on the qualitative aspects of the study was undertaken 22nd March 2018. Twelve people (pregnant women

For peer review only - http://bmjopen.bmj.com/site/about/guidelines.xhtml

n=8 and their birth partners *n*=4) took part. Participants were provided with the Participant Information Leaflet, Consent Form and Interview Topic Guide to review prior to the session. Opinions on being observed during labour and interviewed post birth were specifically sought. There was unanimous agreement that the qualitative research would be accepted and welcomed by women. The opportunity to speak with an obstetrician about the birth on the first postnatal day was viewed as a positive opportunity. No substantial changes to qualitative design of the study were made as a result of this session.

Data collection

The case study investigation included data from one or more of the following sources for each case: participant observation of the attempted Odon-assisted delivery and interviews with women, operators, and midwives. Conducting case studies in the clinical context of device use, with triangulation of data from different sources, generates greater depth of insight through the process of data triangulation, than would be available using other approaches such as serial interviews with women and operators.[32]

Participant observations commenced when the obstetrician entered the woman's room to conduct the AVB and ended when the obstetrician left the room following the birth of the baby. The researcher was a clinical obstetrician and part of the ASSIST Study Team (EJH). Contextual factors, verbal and non-verbal communication were documented on an observation schedule. The observation schedule was developed during preparatory work, prior to the commencement of the ASSIST Study (Supplementary file 1). Observations ceased when no new insights were derived from two consecutive observations. All women

For peer review only - http://bmjopen.bmj.com/site/about/guidelines.xhtml

For peer review only

who had an observed AVB with the Odon Device were invited to participate in an interview on the first postnatal day. The operator and midwife present at a birth using the Odon Device were invited to take part in separate interviews. Interviews were semi-structured and directed by topic guides (Supplementary file 2), which were established based on pre-clinical and clinical literature and developed iteratively during data collection. Each operator and/or midwife could be interviewed more than once during the course of the study as each case was defined by the use of the Odon Device and not the clinicians present. Where possible, all interviews were undertaken face-to-face.

Triangulation of data

Transcripts were created from the participant observations made during the birth. All thoughts and interpretations of the researcher were noted, often aiding the creation of specific interview questions. Any issues that required clarification from participants were highlighted during the transcription process for further discussion in subsequent interviews. Findings were successively tested and refined by the researcher as additional transcripts were analysed and explored in the interviews with participants.

Data analysis

Data collection and analysis ran iteratively and in parallel.[33] NVivo 12 (QSR International, Melbourne, Australia) was used to organise data from all sources as well as supporting analysis. Thematic analysis was used for analysis, using the key steps that have been well described in the literature.[33] A broadly inductive approach to analysis was used, enabling themes to be derived from the data. Triangulation of sources guided the analysis of

For peer review only - http://bmjopen.bmj.com/site/about/guidelines.xhtml

observation data and interview transcripts from a single case to identify, outline, and organise codes. Codes were then grouped together where commonalities were noted to identify initial themes; the description and sample quotes to illustrate the theme were noted. Finally, case summaries were developed and reviewed to create thematic accounts which identified patterns within the data and provided explanations for these. [34]Double coding of a proportion (20%) of interview transcripts was undertaken by JW. Commonality and variances in coding between qualitative researchers were discussed, resolved by consensus and used to further shape developing themes and sampling.

Reflexivity

All case study research, PPI, piloting, data collection and analysis was undertaken by a medically qualified obstetrics and gynaecology trainee, Odon Device trainer and Odon Device operator for the ASSIST Study (EH). The use of the Odon Device to assist birth is highly complex and often takes only minutes to perform; without an in-depth knowledge of the device, its Instructions for Use (IFU) and AVB, subtle differences or anomalies in its use may have overlooked during data collection by a non-clinical professional. It is possible that pre-conceived ideas and suppositions were brought to the study given the researcher's specialist clinical background. To maintain rigour, reflexive comments were made after each data collection event (observation or interview) and during data analysis. These were discussed with an experienced independent qualitative researcher (JW).

Results

For peer review only

226 Presented here is the combination of observational and interview data relevant to women's experiences of the attempted Odon assisted delivery and participation in the intrapartum study. Other data relating to optimising use of the device by operators has been analysed separately and will be published separately.

230 The 39 case studies conducted of attempted Odon assisted births, included eight observations, eight interviews with women observed, 19 midwife interviews, 37 operator interviews and two operator reflections. Demographics and characteristics of the women included are presented in Table 1 and data collected for the 39 case studies in Table 2.

234 **Table 1** Demographics and characteristics of women participating in the case study research compared to those in the ASSIST StudyClick or tap here to enter text.

	**Case studies** n=8(%)	**ASSIST Study** n=40(%)
**Parity**		
Nulliparous	7(87.5)	34(85.0)
Multiparous	1(12.5)	6(15.0)
**Mean age in years** (mean)	28.5	29
**Ethnicity**		
White British	8(100.0)	32(80.0)
Any other white background	0(0.0)	4(10.0)
Black	0(0.0)	2(5.0)
Indian	0(0.0)	1(2.5)
Any other Asian background	0(0.0)	1(2.5)
**Mode of labour onset**		
Spontaneous	0(0.0)	6(15.0)
Induced	8(100.0)	34(85.0)
**Analgesia at time of birth**		
Epidural	4(50.0)	34(85.0)
Perineal infiltration	3(37.5)	12(30.0)
None	1(12.5)	1(2.5)
**Final mode of birth**		
Caesarean section	0(0.0)	2(5.0)
Successful birth with Odon Device	5(62.5)	19(48.0)
Assisted vaginal birth with forceps or ventouse (unsuccessful Odon Device)	3(37.5)	19(48.0)

For peer review only - http://bmjopen.bmj.com/site/about/guidelines.xhtml

Table 2 General information about the case studies (n=39)

	Data collected			
Case study no.	Observation	Interviews		
		Women	Operator	Midwife
1	X O01	X W01	X D02	X M15
2			X D03	X M18
3			X D03	X M12 X M18
4			X D03	X M10
5			X D02	X M18
6	X O02	X W02	X D02	X M19
7	X O03	X W03	X D03	X M11 X M11
8	X O04	X W04	X D02	
9			X D03	X M16
10	X O05	X W05	X D02	X M13
11	X O06	X W06	X D02	X M14
12	X O07	X W07	X D04	X M12
13			X D02	X M16
14			X D03	X M10
15	X O08	X W08	X D03	X M17
16			X D02	
17			X D04	X M20
18			X D06	
19			X D06	
20			X D04	X M11
21			X D06	
22			X D02	X M11
23			X D02	
24			X D06	
25			X D06	
26			X D02	
27			X D02	
28			X R01	
29			X D02	
30			X D06	
31			X D02	
32			X R02	
33			X D03	
34			X D03	
35			X D06	
36			X D02	
37			X D06	
38			X D06	X M11
39			X D04	

For peer review only

Observations varied in length from 33 to 68 minutes. Interviews with women lasted 7 to 10 minutes, interviews with midwives lasted 3 to 13 minutes and interviews with operators lasted between 5 and 26 minutes.

A summary of the themes relating to the women's experiences are presented in Figure 2.

Figure 2 Women's experiences: women's and health professional views- themes and sub-themes.

Theme one: Reasons for taking part – a different assisted birth

All eight women interviewed gave very similar reasons for agreeing to participate in the ASSIST Study and have the Odon Device used to assist the birth of their baby. Women's experiences were predominantly positive even where Odon use had not succeeded.

Half the women interviewed (n=4) stated a specific aversion towards forceps assisted birth as an important factor in their decision making. Midwives shared the antipathy for forceps and the wish for an alternative device and an AVB that women would welcome. Aversion towards forceps was linked to previous negative experiences of friends or family:

> W04: *'And also the forceps, to me, looked like a torture weapon, so I was keen to give something else a go.'*

> W01: *'I don't have a lot of experience of it, but I know my nephew had forceps and was very bruised and marked.'*

For peer review only

Women and midwives articulated similar views, the lay belief that forceps are more 'traumatic' either physically or emotionally:

M14: *'…but it definitely didn't look quite as traumatic as a forceps delivery... But I do feel like, for her, it was a nicer experience than it might have been for a forceps. I don't know if it's the word 'forceps' that traumatises ladies, but everyone seems to be terrified of them.'*

Midwives perceived that the Odon Device was kinder than other devices and described the births as 'softer', 'nicer', 'beautiful', 'easy', 'smooth' and 'gentle':

M18: *'The device use and the delivery was actually kind of one of the most beautiful deliveries of an assisted device used that I've seen.'*

This belief was also reinforced by observations from women, midwives and operators, that babies successfully delivered with the Odon Device had minimal scalp or face injury following birth. The reduction in visual marks on the baby seemed to have a big impact on the overall perception of the birth:

M20: *'These babies are coming out with no marks on them, it's fab. I think it's stunning. It's absolutely really good.'*

W07: *'Over the moon. The fact that it has helped with his delivery, the fact that he's safe, there's no marks on him whatsoever, he's completely fine, and he's just a happy baby. So, that's all I could ever ask for really.'*

For peer review only

Observations highlighted that all staff members present at time of birth, both clinical and research, strove to ensure that the birth experience was positive for women, perhaps more so than in usual clinical practice. Environmental factors seemed to stand out as creating a calm birthing experience:

> O01: *Low lighting in the room, not main lights but two spotlights providing the light to the operator only. Music on in the background was on already prior to entering the room. It was a very calm and quiet environment."*

Team members perceived that birth experience was more positive for women who were felt to have really wanted a birth assisted by the Odon Device:

> M11: '*And then at that point she said, "Oh, come on doctor," she said something like, "I've got faith in you, you can do it." So, she* [the patient] *really, really wanted him* [the operator] *to do it. And then she* [the patient] *said, "But if this doesn't work, I don't want forceps because of the baby's face; I'd rather have a Section."'*

The level of engagement of women and their birth partners with the Odon Device surprised the operators:

> D02: '*… the husband was leaning over her leg and watching exactly what I was doing and was commentating as I was applying the device… So, when I got to four, he was like, "It's on four, it's on four, it's nearly there, nearly there." I wasn't quite sure whether he was encouraging his wife or encouraging me, but it made me smile.'*

For peer review only

Initial generalised altruism regarding research participation was overtaken by genuine engagement and specific interest in the study:

W01: *'I'm quite cynical and I was like, "I'm not going to hear about this again. I just have to say yes to this." Then I read about it and actually I did find it quite interesting, so I was more than happy to sign up to it.'*

Several women stated their surprise when they needed an AVB having assumed during the consenting process that they would not require an assisted birth.

W06: *'It's such a low percentage, isn't it, probability-wise. So, you just, kind of, almost think, "Well, it probably won't come to this. So, might as well [consent]."'*

Women expected and presumed that there would be observations of device use in the ASSIST Study. They appreciated it was important to observe the device in real-life settings and to learn from its use:

W02: *'You're not going to know any different. It's easy saying, "simulations, we'll do these," but until it's gone into practice on a real baby…You need to observe to see how it works…Or how can you, maybe, if something went wrong, fix it. How are you ever going to learn if anything…'*

When asked about the possibility of videoing the AVB instead of having a formal observation by a researcher, views from women were very mixed. For some it was acceptable, providing there was anonymity and that it was used for training, one stated 'no'.

For peer review only - http://bmjopen.bmj.com/site/about/guidelines.xhtml

Theme two: What makes an acceptable recruitment process?

Women valued various aspect of the recruitment process, including provision of information through multimedia format, the manner of the recruiter, timing of when they were approached and treating consent as a process and not a single event, so checking consent multiple times.

The provision of the information video allowed women to visualise and understand how the Odon Device works. They already knew what was going to happen at the time of assisted birth:

> W02: *'…we watched the video. That's what, I think, made us say yes, I suppose, the video. Just, it's quite a good little demo on the video that makes you think…'*

Women preferred this innovative multimedia approach rather than reading the information leaflets:

> W04: *'A leaflet you tend not to read it, do you? And a presentation is dull. So yes, I quite liked the video.'*

They appreciated the opportunity to see the team and operators in the participant video.

The way recruitment was delivered by the research team was also of the upmost important to women. The women described how the manner of checking consent and regular interactions with the research team increased the trust in them:

For peer review only - http://bmjopen.bmj.com/site/about/guidelines.xhtml

W06: *The woman that came round, she had the right approach. You don't want to be too pushy or whatever, and you need to, yes, have tact with people, and she was fine. She was good.*

Research follow-up was experienced as supportive for the women:

W07: *'Even after delivery, just coming in to check and see that everyone's alright, honestly, I didn't find any faults.'*

Theme three: Acceptability of the research process

For the women interviewed, birth experiences were not affected by whether the Odon Device was successful or not or the number of healthcare professionals present at the time of birth.

W02: *'I know, obviously, it wasn't successful, but it's not going to be successful every single time. Everyone's situations are different.' (Unsuccessful Odon birth)*

Interestingly, women focussed on outcomes for their baby and their feelings at the time of birth rather than their own pregnancy outcomes or birth complications when discussing their experiences.

Women could feel overwhelmed at the time of needing an AVB:

For peer review only

O06: *'At the time of needing an AVB, she was losing the plot and scared, she said "help me" "I cannot do this".' (Successful Odon birth)*

Despite this women reported feeling cared for and the situation felt routine. When asked about their birth experience, women reflected on the feeling they had during the birth of their baby:

W07: *'But the device and that, I didn't even make… I know, obviously, it's a big thing, but I didn't even really pay any attention to it. I just thought it was a normal device, like forceps'. (Successful Odon birth)*

Some women ascribed an unsuccessful Odon birth to their baby rather than to device failure. Interestingly, they saw an unsuccessful AVB with the Odon Device as providing useful insights for future understanding and births:

W02: *'I know, obviously, it wasn't successful, but it's not going to be successful every single time. Everyone's situations are different. It was just our baby being difficult. It's different if every single person going didn't like it. But no, I'd, 100%, consent again.' (Unsuccessful Odon birth)*

Some women were surprised that there were increased numbers of staff present at the time of birth but did not state that this affected their birth experience:

W04: *'I must say, it was quite overwhelming when everyone came in at the time the help was needed… They explained they were all for different stuff and for different jobs.' (Successful Odon birth)*

For peer review only - http://bmjopen.bmj.com/site/about/guidelines.xhtml

Half of the women stated that having extra staff present at time of birth was fine as they perceived that the more people, the safer the environment:

> W02: '*I think the more people there, the less you can worry, because you know you're in safe hands…Yes, but you're thinking, "Right, there are 10 people in this room now, so someone's going to be alright."' (Unsuccessful Odon birth)*

Discussion

Main Findings

Qualitative research in the form of a case study investigation into the feasibility of using an innovative device for AVB was used in this study to investigate women's experiences of the attempted Odon-assisted birth and of participating in intrapartum research. This study included observations of attempted AVBs and interviews with women involved, and interviews with midwives and operators to explore women's experiences in detail. Women's experiences were overwhelmingly positive (more so than had been expected by the study team), regardless of the outcome of using the device. Study recruitment and follow-up processes were valued by women and in-person observation of the birth was acceptable.

Women and midwives want an alternative to forceps and ventouse. There are wide inconsistencies in the use of AVB worldwide and these are often multifactorial, relating to concerns regarding complications of AVB and the associated litigation,[5] limited availability of devices in certain settings, public perception,[6] and insufficient training in devices or complex

interventions.[7,12] Our study highlighted a clear maternal aversion towards forceps and a wish for a kinder AVB. There was genuine engagement in research and positive feedback from midwives regarding their perception of the device. Women appreciated the recruitment process and the methodology used for information provision, especially via the participant information video. Providing the women with an information video proved vital in aiding their understanding of the device as well as being a welcome format for receiving information. Women understood the need for qualitative research and expressed an awareness of the need to observe and learn from each assisted birth. Unsuccessful births with the Odon Device were not viewed by women as a failure, rather a learning opportunity for the research team. There was some reservation from midwives regarding the number of people present in the room for an AVB with the Odon Device, especially with formal observation however, it was viewed as acceptable if women were happy. These findings are very similar to a recent systematic review on women's experiences of AVB.[12] As it was the first time the Odon Device had been used in clinically indicated cases[22], there was acceptance and expectation from women that their births be formally observed. There was less evidence that video-recording the births was acceptable to women.

Strengths and Limitations

This is the first study to undertake an in-depth qualitative exploration of the Odon Device for clinically indicated AVBs. Capturing the experiences of women is vital in assessing the device in clinical trials. Conducting case studies on the labour ward enabled researchers to gather a 'genuine' representation of experience within the environment in which the device is used that might not have been achieved using alternative approaches.[32] Another key advantage of gathering data from multiple sources (participant observation and interviews)

For peer review only - http://bmjopen.bmj.com/site/about/guidelines.xhtml

is the opportunity for triangulation of a wealth of data. Data amalgamation (within cases) combined with constant cross case comparison, improved the validity of the study findings. A further strength is that where there are indications that saturation has been reached, there is an opportunity for one form of data collection to be dropped, applied here to observation data. Reporting was undertaken following the Standards for Reporting Qualitative Research[34] (Supplementary information 3).

Despite these advantages, there are limitations to this study. Only women accepting participation in ASSIST were observed or interviewed and those declining participation may have different views. There were only eight case studies involving observation and interviews with women, because data saturation was reached from observations. This limitation raises the possibility that findings are less generalisable. Furthermore, the interviews with women were short. There are several reasons as to why this may be the case. Firstly, women were only asked a very small number of questions specifically relating to their birth experiences. Operators and midwives however were asked questions not only on experience but device use, technique, design and trial methodology. Additionally, women were interviewed at day one postnatal, when likely fatigued. This was discussed during the PPI sessions and women agreed that interviewing women in hospital close to the birth was more suitable than later at home, as women were aware that their memory of their birth experience may not be reliable. The case studies were undertaken by a specialist trainee in obstetrics and gynaecology meaning that pre-conceptions and existing knowledge may have influenced the collection and interpretation of the data. However, at the time of commencing the case studies the researcher was naïve to the use of the Odon Device in the clinical setting. Lastly, operators may have changed their behaviours during observations,

For peer review only

perhaps not reflecting their real-life practice. It is possible that there was response bias as women knew that the researcher was a clinician in the trial. However, this was discussed during PPI and valued as an attraction of the study for women. Women and their partners were keen to discuss their birth with a clinician who knew the device, feeling it was an essential aspect of the research process.

For peer review only

Interpretation

With novel devices being tested in clinical trials, it is vital that experiences of key stakeholders are explored. Having an innovative device for AVB that has high clinical efficacy is not useful if women find it unacceptable. Our findings suggest that women are willing to accept an alternative device for AVB, especially one that is perceived as kinder to their babies. Women commended the use of video during the recruitment process and expressed support for intrapartum research.

Conclusion

This study demonstrates that qualitative case study methodology enables the detailed exploration of experiences and views of women and healthcare professionals in intrapartum research and that the methods (especially observation) were acceptable and expected by women.

When introducing or implementing a novel device into clinical use it is vital that the experiences and opinions of stakeholders are considered. Case studies provide a way of exploring these views in detail by integrating data from multiple sources, providing a

For peer review only - http://bmjopen.bmj.com/site/about/guidelines.xhtml

comprehensive and authentic exploration. Case study methodology is used widely in other avenues of research however it has not yet been widely adopted in implementation research during device or surgical trials.[35] Successful completion of case studies on labour ward for an intrapartum intervention has provided methodology that can be transferred to intervention delivery and design of novel devices. Women appear keen to support the investigation of novel devices for AVB, such as the Odon Device, and value the importance of information sharing during the recruitment process. The perception of women can help shape and design future recruitment strategies, information provision and intervention delivery. The use of case study methodology must be further tested in alternative settings and specialties.

Acknowledgements

The authors would like to thank all the women who agreed to take part in this research and all the maternity staff who enabled to safe completion of the ASSIST Study.

Competing interests

EL is a member of University of Bristol (UoB) and part of his salary is paid by PROMPT Maternity Foundation (PMF); PMF has received funding from a Saving Lives at Birth award via a subcontract from Becton, Dickinson and Company (BD) to conduct preclinical simulation studies of the Odon Device, these funds have been used towards the salary of

For peer review only

TJD and JFC when undertaking the simulations studies. All other authors report no competing interests.

Author contributions

EJH, NSB and JW developed the concept for the qualitative research. EJH performed all data collection and analysis with co-coding performed by JW. EJH wrote the initial draft of the manuscript with support from NSB, JFC and JW. EJH, NSB, EL, TJD, JFC and JW reviewed and approved the final manuscript.

Ethical approval

The research was approved by South Central–Berkshire REC, UK on 3rd September 2018 (18/SC/0344), the MHRA on 9th August 2018 and the HRA on 3rd September 2018.

Data sharing statement: Data are available upon reasonable request.

Funding

This research was supported by the Bill & Melinda Gates Foundation [OPP1184825/INV-010180]. The Foundation had no role in study design, planning, conduct, analysis or publication production.

For peer review only

References

1. Lawn JE, Blencowe H, Waiswa P, Amouzou A, Mathers C, Hogan D, et al. Stillbirths: rates, risk factors, and acceleration towards 2030. Lancet. 2016;387(10018):587–603.

2. (UNIGME) UNIG for CME. Levels & Trends in Child Mortality: Report 2019, Estimates developed by the United Nations Inter-agency Group for Child Mortality Estimation [Internet]. 2019 [cited 2021 Oct 11]. Available from: https://www.unicef.org/reports/levels-and-trends-child-mortality-report-2020

3. WHO. Trends in maternal mortality: 2000 to 2017: estimates by WHO, UNICEF, UNFPA, World Bank Group and the United Nations Population Division. [Internet]. 2019 [cited 2021 Oct 11]. Available from: https://www.unfpa.org/featured-publication/trends-maternal-mortality-2000-2017

4. WHO, UNICEF, AMDD, UNFPA. Monitoring emergency obstetric care: a handbook [Internet]. WHO; 2009 [cited 2021 Oct 11]. Available from: http://whqlibdoc.who.int/publications/ 2009/9789241547734_eng.pdf?ua=1

5. Murphy DJ. Medico-legal considerations and operative vaginal delivery. Best Pract Res Clin Obstetrics Gynaecol. 2019;56:114–24.

6. J F. Why do doctors still use forceps when they killed out baby? Daily Mail [Internet]. 2010 Feb 22;1–36. Available from: https://www.dailymail.co.uk/health/article-1253013/Forceps-killed-baby-doctors-using-them.html

7. Gale A, Siassakos D, Attilakos G, Winter C, Draycott T. Operative vaginal birth: better training for better outcomes. BJOG. 2014;121(5):643–4.

8. Murphy DJ, Liebling RE, Verity L, Swingler R, Patel R. Early maternal and neonatal morbidity associated with operative delivery in second stage of labour: a cohort study. Lancet. 2001;358(9289):1203–7.

9. RCOG. Operative Vaginal Delivery: Green–top Guideline No. 26 [Internet]. 2011. Available from: https://www.rcog.org.uk/globalassets/documents/guidelines/gtg_26.pdf

10. Mobbs N, Williams C, Weeks A. Humanising birth: Does the language we use matter? [Internet]. 2018 [cited 2021 Oct 11]. Available from: http://blogs.bmj.com/bmj/2018/02/08/humanising-birth-does-the-language-we-use-matter/

11. NICE. Intrapartum care for healthy women and babies [Internet]. 2014. Available from: https://www.nice.org.uk/guidance/cg190/resources/intrapartum-care-for-healthy-women-and-babies-pdf-35109866447557

For peer review only

12. Crossland N, Kingdon C, Balaam M-C, Betrán AP, Downe S. Women's, partners' and healthcare providers' views and experiences of assisted vaginal birth: a systematic mixed methods review. Reprod Health. 2020;17(1):83.

13. Weiner C, Samuelson L, Collins L, Satterwhite C. 5-year experience with PROMPT (PRactical Obstetric Multidisciplinary Training) reveals sustained and progressive improvements in obstetric outcomes at a US hospital. Am J Obstet Gynecol. 2014;210(1):S40.

14. Siassakos D, Fox R, Hunt L, Farey J, Laxton C, Winter C, et al. Attitudes Toward Safety and Teamwork in a Maternity Unit With Embedded Team Training. Am J Med Qual. 2011;26(2):132–7.

15. Aggarwal R, Mytton OT, Derbrew M, Hananel D, Heydenburg M, Issenberg B, et al. Training and simulation for patient safety. BMJ Quality & Safety. 2010;19(Suppl 2):i34–43.

16. Hotton E, O'Brien S, Draycott TJ. Skills training for operative vaginal birth. Best Pract Res Clin Obstet Gynaecol. 2018;56:11–22.

17. Malmstrom T. The vacuum extractor, an obstetrical instrument. I. Acta obstetricia et gynecologica Scandinavica. 1957;36:5–50.

18. O'Brien SM, Mouser A, Odon JE, Winter C, Draycott TJ, Sumitro T, et al. Design and development of the BD Odon DeviceTM : a human factors evaluation process. BJOG: An International Journal of Obstetrics & Gynaecology. 2017;124 Suppl 4:35–43.

19. O'Brien SM, Winter C, Burden CA, Boulvain M, Draycott TJ, Crofts JF. Fetal head position and perineal distension associated with the use of the BD Odon Device™ in operative vaginal birth: a simulation study. BJOG: An International Journal of Obstetrics & Gynaecology. 2017 Sep;124(11):10–8.

20. O'Brien SM, Winter C, Burden CA, Boulvain M, Draycott TJ, Crofts JF. Pressure and traction on a model fetal head and neck associated with the use of forceps, Kiwi™ ventouse and the BD Odon Device™ in operative vaginal birth: a simulation study. BJOG. 2017;124(11):19–25.

21. Lange C de, Saugstad OD, Solberg R. Assessment of cerebral perfusion with contrast-enhanced ultrasound during constriction of the neck mimicking malposition of the BD Odon Device™: a study in newborn piglets. BJOG: An International Journal of Obstetrics & Gynaecology. 2017 Sep;124 Suppl 4(4 Pt 1):26–34.

22. Hotton EJ, Lenguerrand E, Alvarez M, O'Brien S, Draycott TJ, Crofts JF, et al. Outcomes of the novel Odon Device in indicated operative vaginal birth. Am J Obstet Gynecol. 2021;224(6):e607.

23. Atchan M, Davis D, Foureur M. A methodological review of qualitative case study methodology in midwifery research. Journal of advanced nursing. 2016;72(10):2259–71.

For peer review only - http://bmjopen.bmj.com/site/about/guidelines.xhtml

24. Bick DE, Rycroft-Malone J, Fontenla M. A case study evaluation of implementation of a care pathway to support normal birth in one English birth centre: anticipated benefits and unintended consequences. BMC pregnancy and childbirth. 2009;9:47.

25. Sittner BJ, DeFrain J, Hudson DB. Effects of high-risk pregnancies on families. MCN, The American Journal of Maternal/Child Nursing. 2005;30(2):121–6.

26. Ericson J, Anagrius C, Rygaard A, Guntram L, Wendel SB, Hesselman S. Women's experiences of receiving information about and consenting or declining to participate in a randomized controlled trial involving episiotomy in vacuum-assisted delivery: a qualitative study. Trials. 2021;22(1):658.

27. Lawton J, Snowdon C, Morrow S, Norman JE, Denison FC, Hallowell N. Recruiting and consenting into a peripartum trial in an emergency setting: a qualitative study of the experiences and views of women and healthcare professionals. Trials. 2016 Apr 11;17(1):195.

28. O'Brien S, Hotton EJ, Lenguerrand E, Winter C, Draycott TJ, Crofts JF, et al. The ASSIST Study - The BD Odon Device for assisted vaginal birth: a safety and feasibility study. Trials. 2019;20(1).

29. Bruni A. Shadowing Software and Clinical Records: On the Ethnography of Non-Humans and Heterogeneous Contexts. Organization [Internet]. 2005;12(3):357–78. Available from: http://journals.sagepub.com/doi/10.1177/1350508405051272

30. Koerber A, McMichael L. Qualitative Sampling Methods. Journal of Business and Technical Communication. 2008 Oct;22(4):454–73.

31. Reeves S, Kuper A, Hodges BD. Qualitative research methodologies: ethnography. BMJ (Clinical research ed). 2008;337:a1020.

32. Rowley J. Using case studies in research. Management Res News. 2002;Volume 25(Issue 1):16–27.

33. Braun V, Clarke V. Using thematic analysis in psychology. Qualitative Research in Psychology. 2006;3(2):77–101.

34. O'Brien BC, Harris IB, Beckman TJ, Reed DA, Cook DA. Standards for Reporting Qualitative Research: A Synthesis of Recommendations. Acad Med. 2014;89(9):1245–51.

35. Blencowe NS, Blazeby JM, Donovan JL, Mills N. Novel ways to explore surgical interventions in randomised controlled trials: applying case study methodology in the operating theatre. Trials. 2015;16:589.

For peer review only

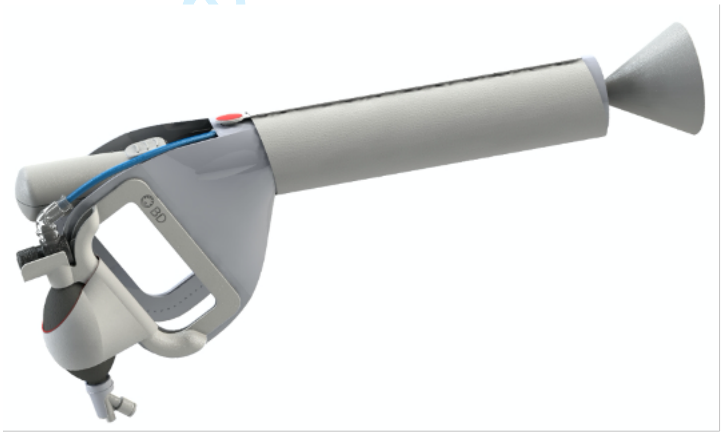

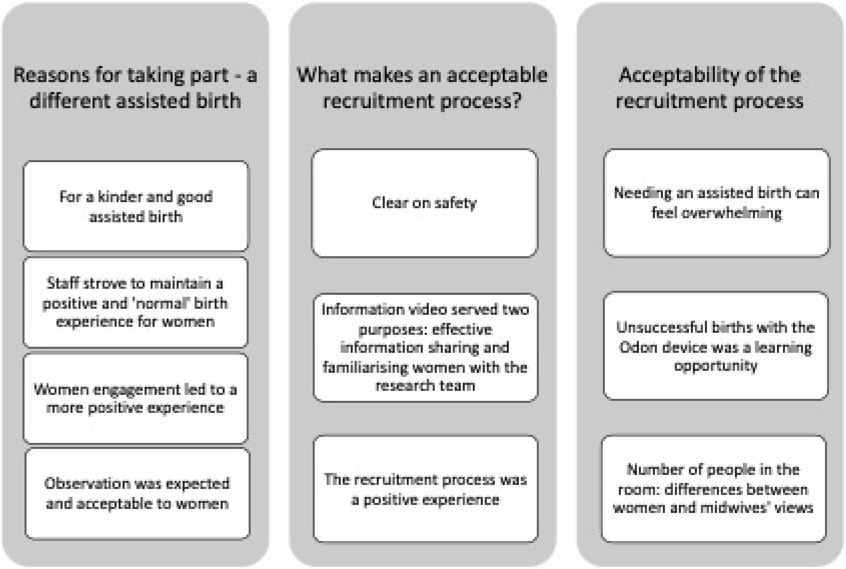

Women's experiences: women's and health professional views- themes and sub-themes

159x110mm (72 x 72 DPI)

North Bristol NHS
NHS Trust

ASSIST
ASSISTED VAGINAL BIRTH STUDY

Patient ID_ _ _ _ Doctor ID _ _ _ _

OBSERVATIONAL DATA SCHEME

**Doctor consent verbally reconfirmed:**	**Yes** ☐	**Date:** **Print:**	**Time:** **Sign**
**Location**		**Operator Grade**	
**Start time of observation**		**End time of observation**	

**Who's present (circle)**	Operator<br>Trainee O&G<br>Consultant O&G	Midwife<br>Student midwife<br>MCA	Anaesthetist<br>Trainee anaesthetist<br>ODP	Scrub nurse<br>Runner<br>Other:

**Pre-delivery**	
Analgesia<br>Birthing partners<br>Consent	
**Delivery**	
Physical<br>People<br>Patient factors<br>Baby factors<br>Lighting<br>Equipment<br>Factors if BD Odon device fails?	
**Post-delivery**	
Catheter?<br>PPH?	

Observation data scheme v4 (03/06/18)

For peer review only

North Bristol NHS
NHS Trust

ASSIST
ASSISTED VAGINAL BIRTH STUDY

Stage	Instruction for use steps	
**Conditions of safe application**	Full Dilation<br>Head 0-+3 station<br>Cephalic presentation confirmed<br>Rupture of membranes confirmed	
	Adequate analgesia	
	Lithotomy position	
	Empty bladder	
	Re-confirm fetal position	
	Lubricate birth canal	
**Device Preparation 1**	Remove device from packaging, maintaining sterility	
	Pull back on fastening band until blue deflation line exposed	
	With fastening band still intact, generously lubricate the inside and outside of the sleeve and the cup	
	While holding the applicator handle gently slide the fastening band back to the top of the sleeve	
	Grip the applicator handle and ensure the viewing window is facing upwards	
**Device Insertion**	Fold the cup and gently insert it through the vulva	
	Check it has regained its circular shape	
	Check there is no maternal tissue trapped between the cup and the fetal head	
	With your hand on the handle of the applicator, gently push the device through the vulva	
	Use your other hand to guide the device and do not apply any force with it	
**Device Preparation 2**	Unfasten the red button	
	Open and completely remove the fastening band	
	Ensure the sleeve and applicator remain in place inside the vulva	
	Between contraction, keeping both hands away from the sleeve, continue to gently push the applicator	
	Starting at 45 degrees below the horizontal and following the curvature of the birth canal	
	Monitor progress by looking through the viewing window	
	Continue to insert the device and stop when "0" appears in the viewing window	
	Squeeze the bulb pump fully and firmly at least 8 times to inflate the cuff	
	Whilst protecting the perineum with one hand, use the other hand to completely withdraw the applicator and cuff, leaving on the sleeve in place	
	To compensate for the possible reduction in cuff pressure, squeeze the bulb pump fully and firmly two more times prior to traction	
**Delivery**	Grasp the sleeve handle, and during contractions pull gently and progressively following the J-shape of the birth canal	
	While continuing to pull gently along the J-shape of the birth canal, confirm the fetal head is descending with pulling efforts	
	If baby has not been delivered with the first pull, repeat the above three steps with any subsequent contractions	
	Once you see the blue deflation line completely, deflate the cuff by pushing on the blue deflation button	
	Pull the sleeve handle continuing to press in the deflation button following the J-shape of the birth canal	
	Continue to pull the sleeve while pressing the blue deflation button to pull the fetal head until the sleeve detaches from the head	
	Proceed to assist the birth of the baby as per normal procedure	
	Discard the disposable applicator and sleeve according to local procedure	
**Extra Steps**	Episiotomy<br>Other: need for second device	

Observation data scheme v4 (03/06/18)

For peer review only - http://bmjopen.bmj.com/site/about/guidelines.xhtml

Investigate patient experience of the study and case studies
Investigate patient experience of the device
Investigate patient view on device monitoring in trials

North Bristol NHS
NHS Trust

ASSIST
ASSISTED VAGINAL BIRTH STUDY

ASSIST STUDY TOPIC GUIDE - WOMEN

Setting: within maternity department, Southmead hospital
Participants: women who have had an AVB using the Odon device

Opening: Interviewer will re-iterate study information, answer any questions, and re-confirm consent.

Background: interviewee details and icebreaker

- Enquire about the baby (name etc.)
- Enquire about their stay in hospital so far
- Enquire about any previous deliveries (NVD, assisted birth etc.)

What has your experience been of being in the study so far?

- Opportunity to reflect on any observations if relevant

What was your reaction when invited to take part?

Probe:

- *Views on the recruitment process?*
- *How did you react when you were asked to have your delivery observed?*
- *How did you react when you were asked to be interviewed? What was your reaction…..?*

How was it being observed?

How do you feel about your birth experience?
How do you feel about the device that was used to help deliver your baby?

Probe:

- *Would they consent to the device being used again? Or not? Why?*
- *Would they recommend it to other women in a similar situation who needed assistance to delivery their baby?*
- *Comparison to other assisted births (if applicable)*

This is a new device, we want doctors using the device to use it at a certain standard. How do you feel about your doctor being observed delivering your baby using the device?

Probe:

- *Why is it alright?*
- *Why is it not alright?*

Closing: interviewer checks understanding of any outstanding points, answers further questions.

Patient interview topic guide v1 (03/06/18)

For peer review only - http://bmjopen.bmj.com/site/about/guidelines.xhtml

Table S3 Standards for Reporting Qualitative Research (SRQR)

No.	Topic	Page number in manuscript
	**Title and abstract**	
S1	Title	1
S2	Abstract	2-3
	**Introduction**	
S3	Problem formulation	5-6
S4	Purpose or research question	6
	**Methods**	
S5	Qualitative approach and research paradigm	7
S6	Research characteristics and reflexivity	10
S7	Context	7
S8	Sampling strategy	7
S9	Ethical issues pertaining to human subjects	11
S10	Data collection methods	8
S11	Data collection instruments and technologies	8
S12	Units of study	10-11
S13	Data processing	7-9
S14	Data analysis	9
S15	Techniques to enhance trustworthiness	8-10
	**Results/findings**	
S16	Synthesis and interpretation	10-18
S17	Links to empirical data	10-18
	**Discussion**	
S18	Integration with prior work, implications, transferability, and contribution(s) to the field	18-22
S19	Limitations	20
	**Other**	
S20	Conflicts of interest	23
S21	Funding	24

For peer review only

No.	Topic	Item
	**Title and abstract**	
S1	Title	Concise description of the nature and topic of the study Identifying the study as qualitative or indicating the approach (e.g., ethnography, grounded theory) or data collection methods (e.g., interview, focus group) is recommended
S2	Abstract	Summary of key elements of the study using the abstract format of the intended publication; typically includes background, purpose, methods, results, and conclusions
	**Introduction**	
S3	Problem formulation	Description and significance of the problem/phenomenon studied; review of relevant theory and empirical work; problem statement
S4	Purpose or research question	Purpose of the study and specific objectives or questions
	**Methods**	
S5	Qualitative approach and research paradigm	Qualitative approach (e.g., ethnography, grounded theory, case study, phenomenology, narrative research) and guiding theory if appropriate; identifying the research paradigm (e.g., postpositivist, constructivist/ interpretivist) is also recommended; rationale[b]
S6	Researcher characteristics and reflexivity	Researchers' characteristics that may influence the research, including personal attributes, qualifications/experience, relationship with participants, assumptions, and/or presuppositions; potential or actual interaction between researchers' characteristics and the research questions, approach, methods, results, and/or transferability
S7	Context	Setting/site and salient contextual factors; rationale[b]
S8	Sampling strategy	How and why research participants, documents, or events were selected; criteria for deciding when no further sampling was necessary (e.g., sampling saturation); rationale[b]
S9	Ethical issues pertaining to human subjects	Documentation of approval by an appropriate ethics review board and participant consent, or explanation for lack thereof; other confidentiality and data security issues
S10	Data collection methods	Types of data collected; details of data collection procedures including (as appropriate) start and stop dates of data collection and analysis, iterative process, triangulation of sources/methods, and modification of procedures in response to evolving study findings; rationale[b]
S11	Data collection instruments and technologies	Description of instruments (e.g., interview guides, questionnaires) and devices (e.g., audio recorders) used for data collection; if/how the instrument(s) changed over the course of the study
S12	Units of study	Number and relevant characteristics of participants, documents, or events included in the study; level of participation (could be reported in results)
S13	Data processing	Methods for processing data prior to and during analysis, including transcription, data entry, data management and security, verification of data integrity, data coding, and anonymization/deidentification of excerpts
S14	Data analysis	Process by which inferences, themes, etc., were identified and developed, including the researchers involved in data analysis; usually references a specific paradigm or approach; rationale [b]
S15	Techniques to enhance trustworthiness	Techniques to enhance trustworthiness and credibility of data analysis (e.g., member checking, audit trail, triangulation); rationale[b]
	**Results/findings**	
S16	Synthesis and interpretation	Main findings (e.g., interpretations, inferences, and themes); might include development of a theory or model, or integration with prior research or theory
S17	Links to empirical data	Evidence (e.g., quotes, field notes, text excerpts, photographs) to substantiate analytic findings
	**Discussion**	
S18	Integration with prior work, implications, transferability, and contribution(s) to the field	Short summary of main findings; explanation of how findings and conclusions connect to, support, elaborate on, or challenge conclusions of earlier scholarship; discussion of scope of application/ generalizability; identification of unique contribution(s) to scholarship in a discipline or field
S19	Limitations	Trustworthiness and limitations of findings
	**Other**	
S20	Conflicts of interest	Potential sources of influence or perceived influence on study conduct and conclusions; how these were managed
S21	Funding	Sources of funding and other support; role of funders in data collection, interpretation, and reporting

[a]The authors created the SRQR by searching the literature to identify guidelines, reporting standards, and critical appraisal criteria for qualitative research; reviewing the reference lists of retrieved sources; and contacting experts to gain feedback. The SRQR aims to improve the transparency of all aspects of qualitative research by providing clear standards for reporting qualitative research.

[b]The rationale should briefly discuss the justification for choosing that theory, approach, method, or technique rather than other options available, the assumptions and limitations implicit in those choices, and how those choices influence study conclusions and transferability. As appropriate, the rationale for several items might be discussed together.

ACADEMIC MEDICINE
